# Supplementary figures and images for: Distinct microglial transcriptomic signatures within the hippocampus
Source: PLoS One. 2024 Jan 5;19(1):e0296280. doi: 10.1371/journal.pone.0296280 (PMC10775894; doi:10.1371/journal.pone.0296280)

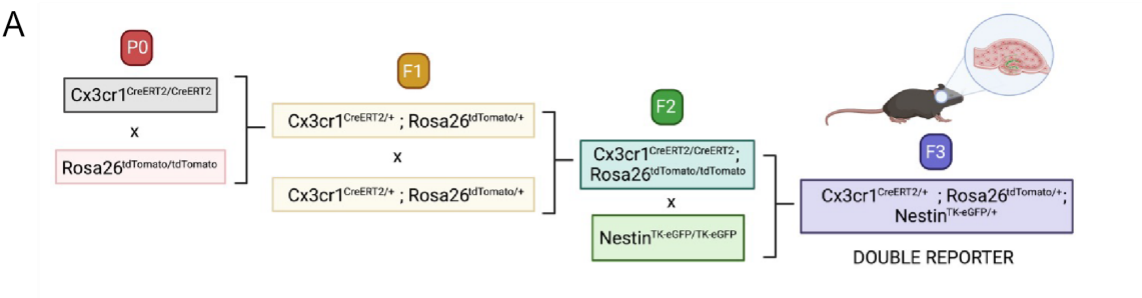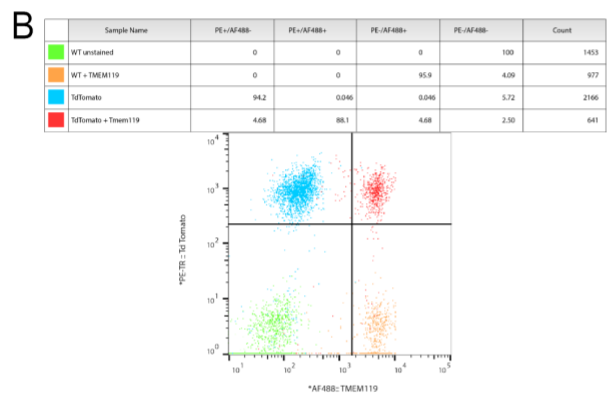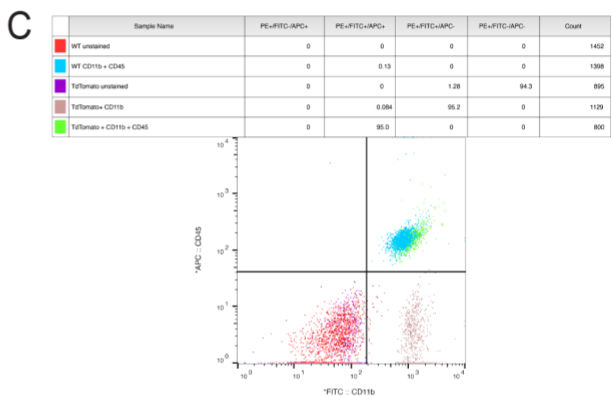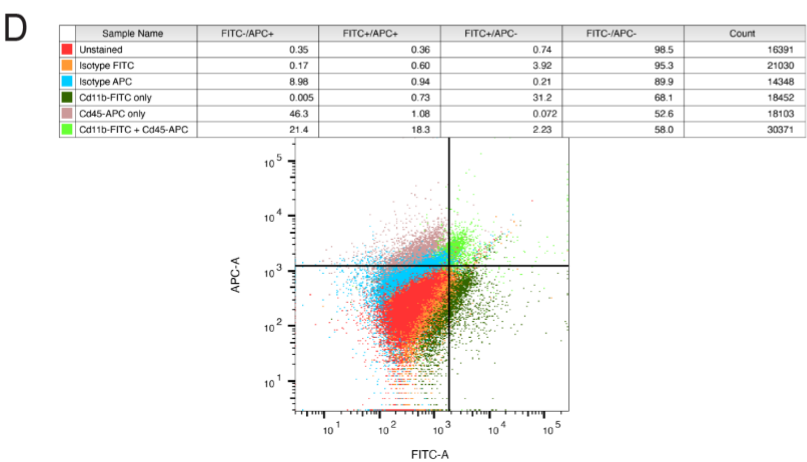

Supplement: S1 Fig — A. Generation of double reporter mouse line. Schematic displaying breeding schemes and selection of breeders to generate mice expressing eGFP in Nestin+ neural progenitor cells and tdTomato in cells from fractalkine (Cx3Cr1) expressing cells and their daughter cells. B. Percentage of cells showing dual expression of Tmem119 and tdTomato, Tmem119 only, TdTomato only, or neither. Results are shown for samples derived from transgenic mouse or wildtype mouse (C57/6Bl). “Count” column contains number of cells per sample recorded. C. Percentage of TdTomato+ cells with CD11B and CD45 expression. Unstained samples (WT unstained, TdTomato unstained) are negative controls for antibody staining. D. Isotype control stainings to show nonspecific background binding for FITC and APC conjugated antibodies. (PDF) [file pone.0296280.s001.pdf]

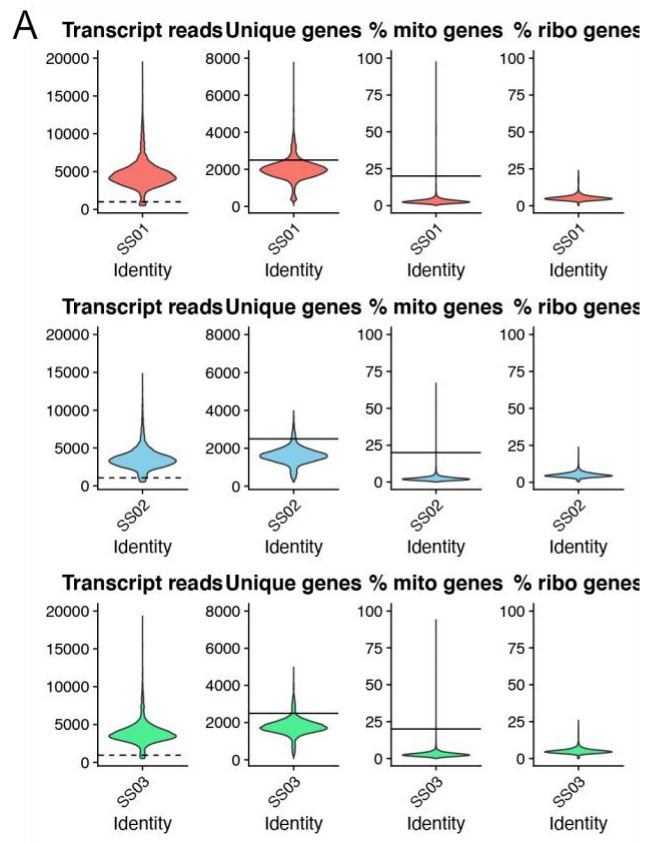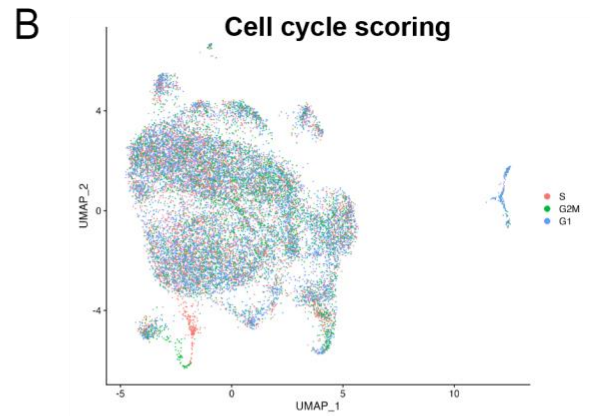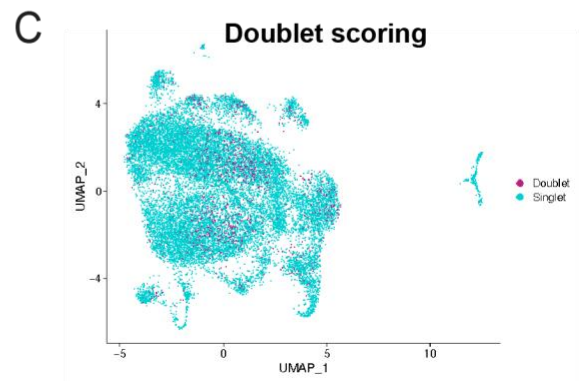

Supplement: S2 Fig — (A) Violin Plots displaying transcript reads, number of unique genes, percentage of reads corresponding to mitochondrial genes, and percentage of reads corresponding to ribosomal genes in each sequencing sample. Dashed line corresponds to lower limit for filtering cells, solid lines represent maximum values for filtering. (B) UMAP plot displaying cell cycle scoring for cells in dataset. (C) UMAP plot displaying predicted doublets based on DoubletFinder. Predicted doublets are highlighted in magenta. (PDF) [file pone.0296280.s002.pdf]

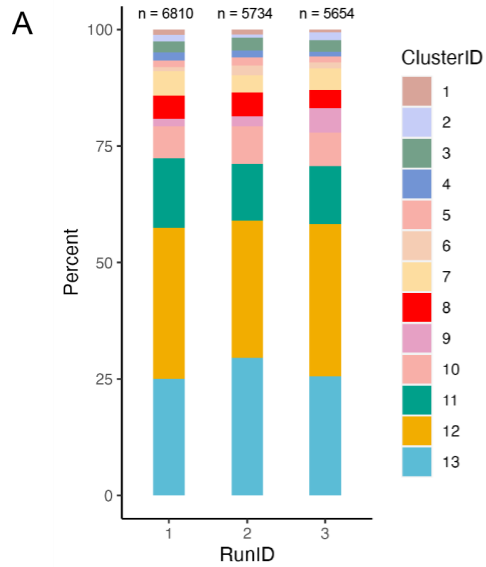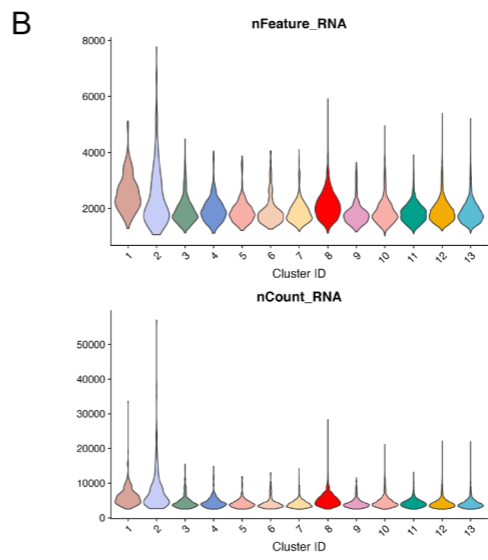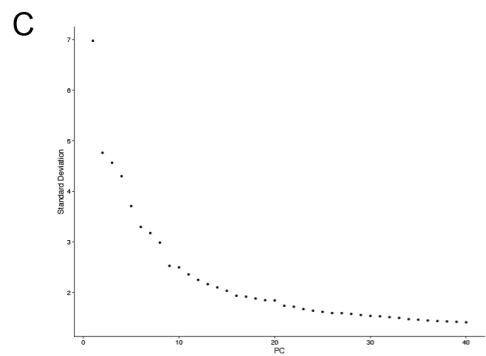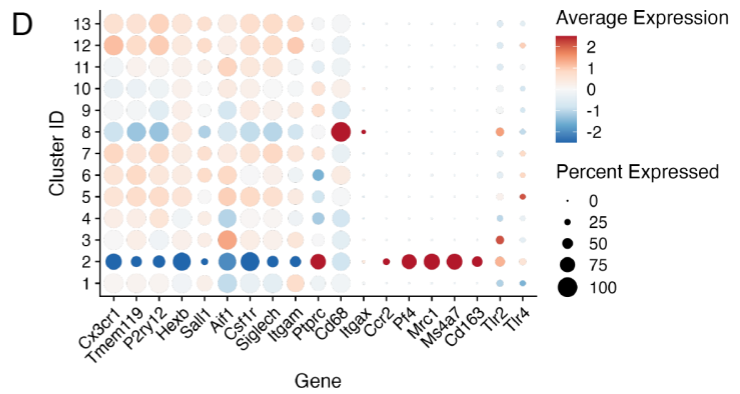

Supplement: S3 Fig — A. Breakdown of subpopulation frequencies within clusters. B. QC violin plots showing number unique genes (nFeature_RNA) and number of reads (nCount_RNA) in each cluster. C. Elbow plot showing variance represented by principal components. D. Dot Plot of myeloid lineage marker genes. (PDF) [file pone.0296280.s003.pdf]

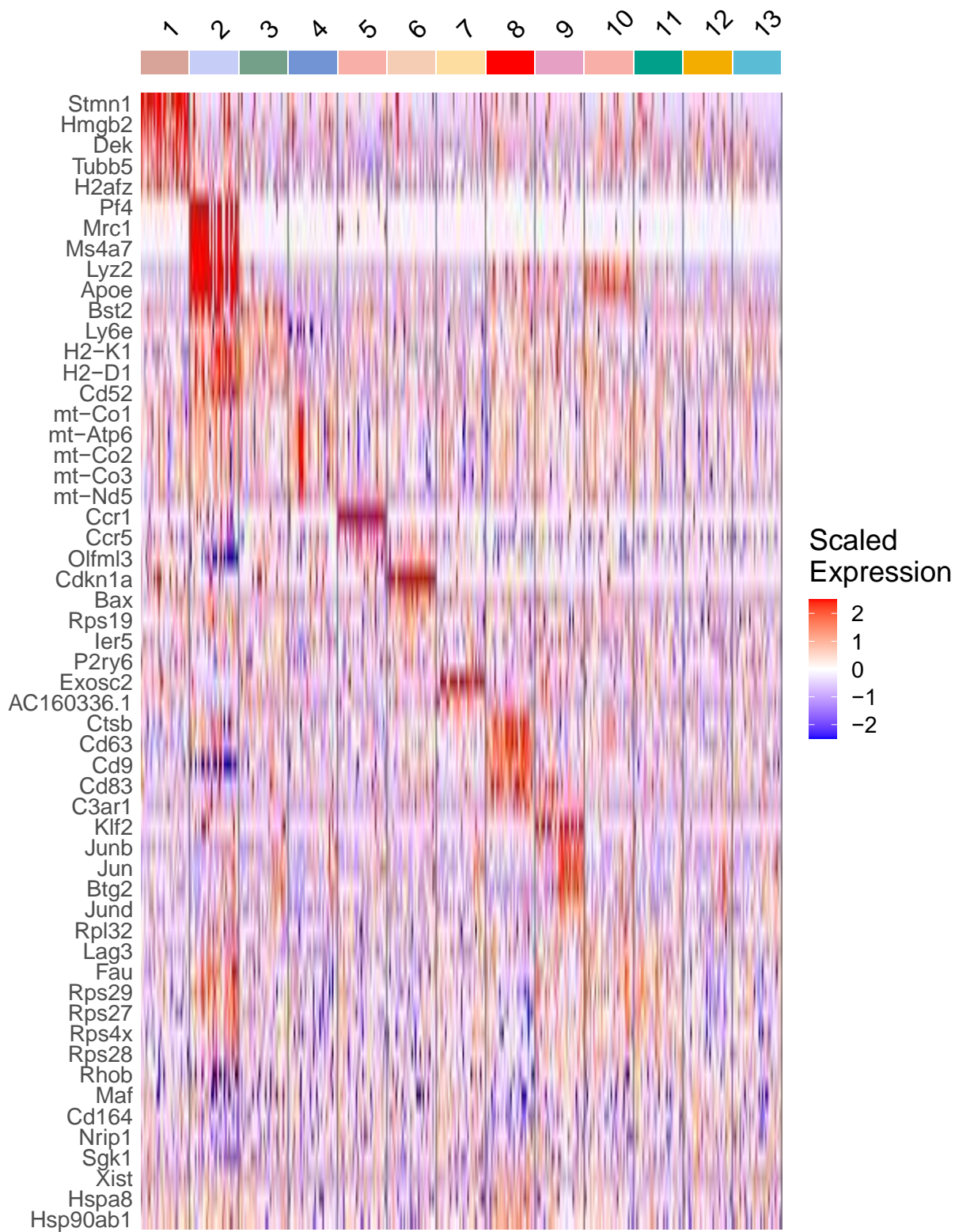

Supplement: S4 Fig — Thirty cells were sampled for each cluster. Each vertical line represents scaled z-score value of gene expression across row. (PDF) [file pone.0296280.s004.pdf]

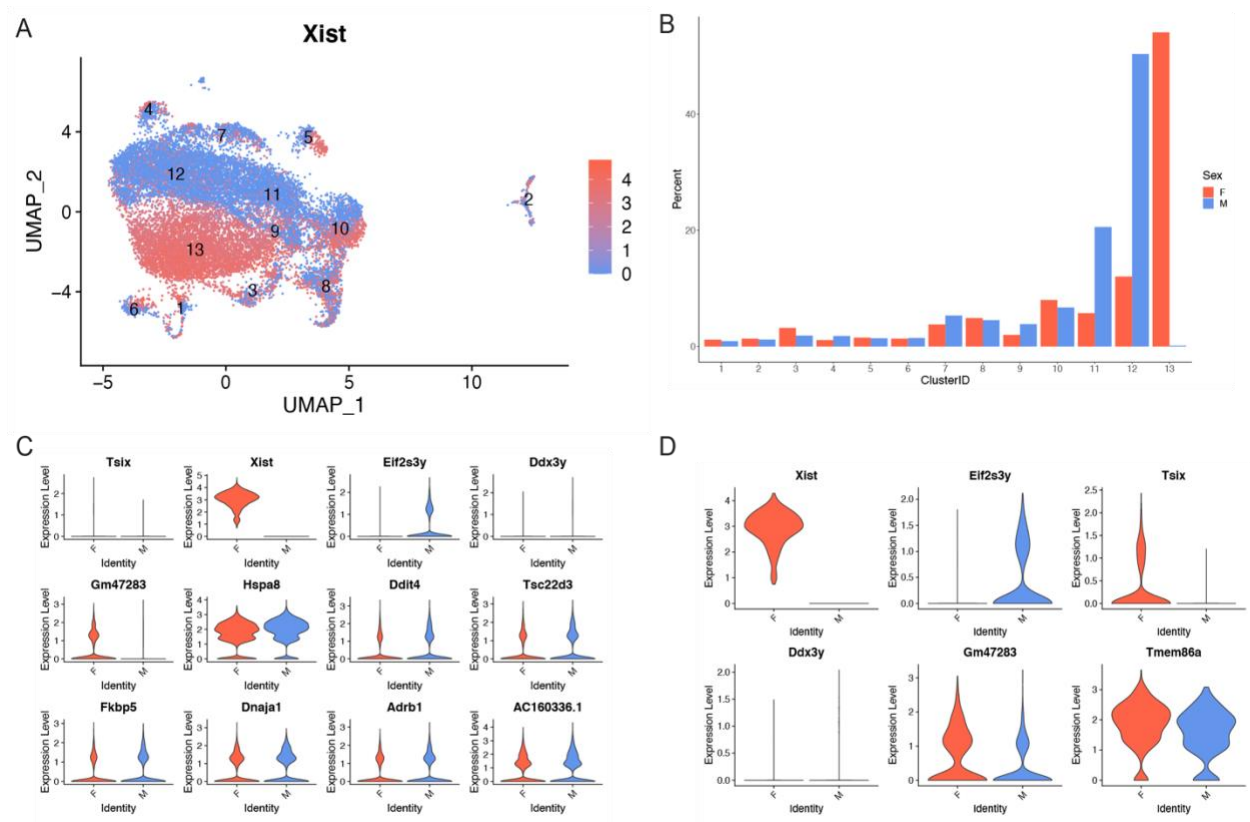

Supplement: S5 Fig — (A) Feature plot displaying scaled expression of Xist projected onto UMAP. (B) Distribution of cells Xist+ female cells versus Xist—male cells across clusters. (C) Violin Plots displaying genes differentially expressed between bulk female (F) and Male (M) cells. (D) Violin plots displaying genes differentially expressed between female and male cells in SGZ cluster 8. (PDF) [file pone.0296280.s005.pdf]

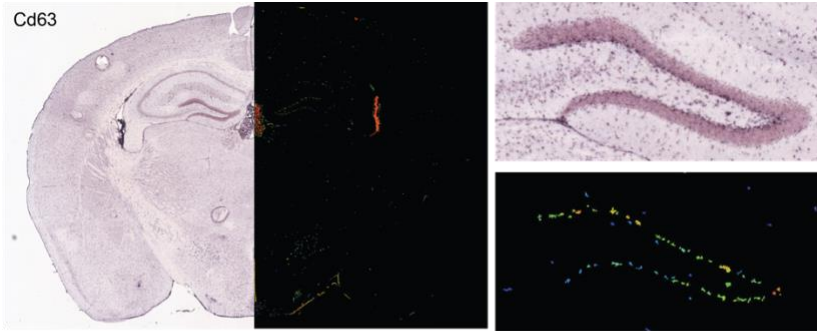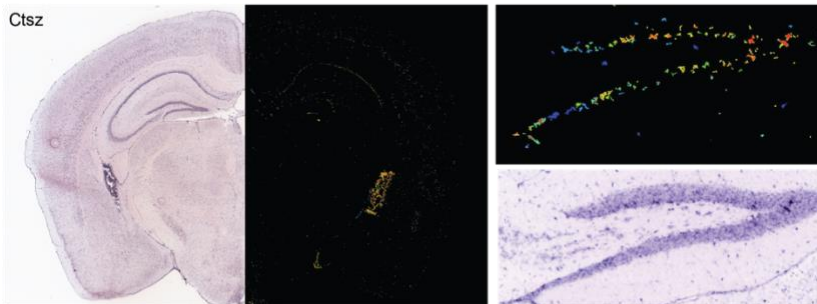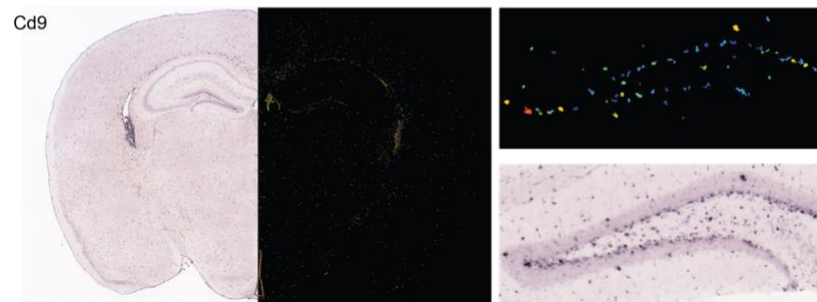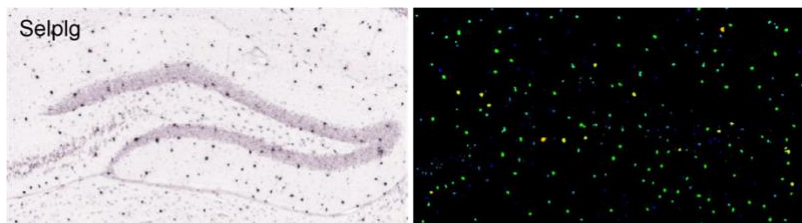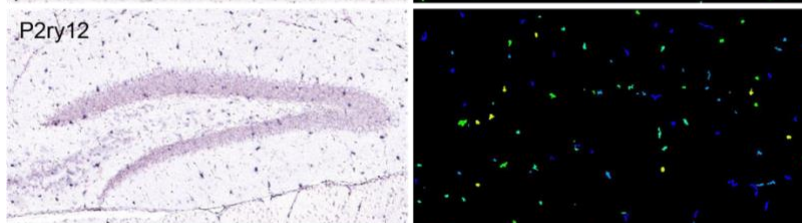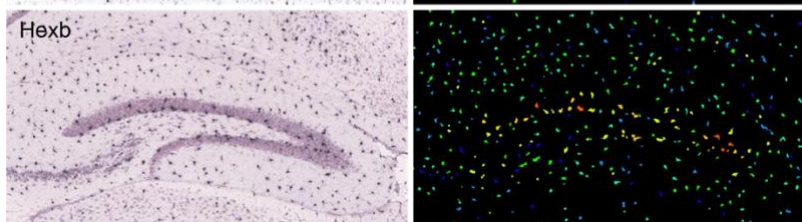

Supplement: S6 Fig — In situ hybridization images from the Allen Institute of genes enriched in cluster 8 (top three) and marker genes (Hexb) or genes associated with homeostatic microgglia (Selplg, P2ry12). For enriched genes, hemicortices (left) are shown to display level of specificity of these genes in the SGZ with more zoomed in fields of view alongside them (right). (PDF) [file pone.0296280.s006.pdf]

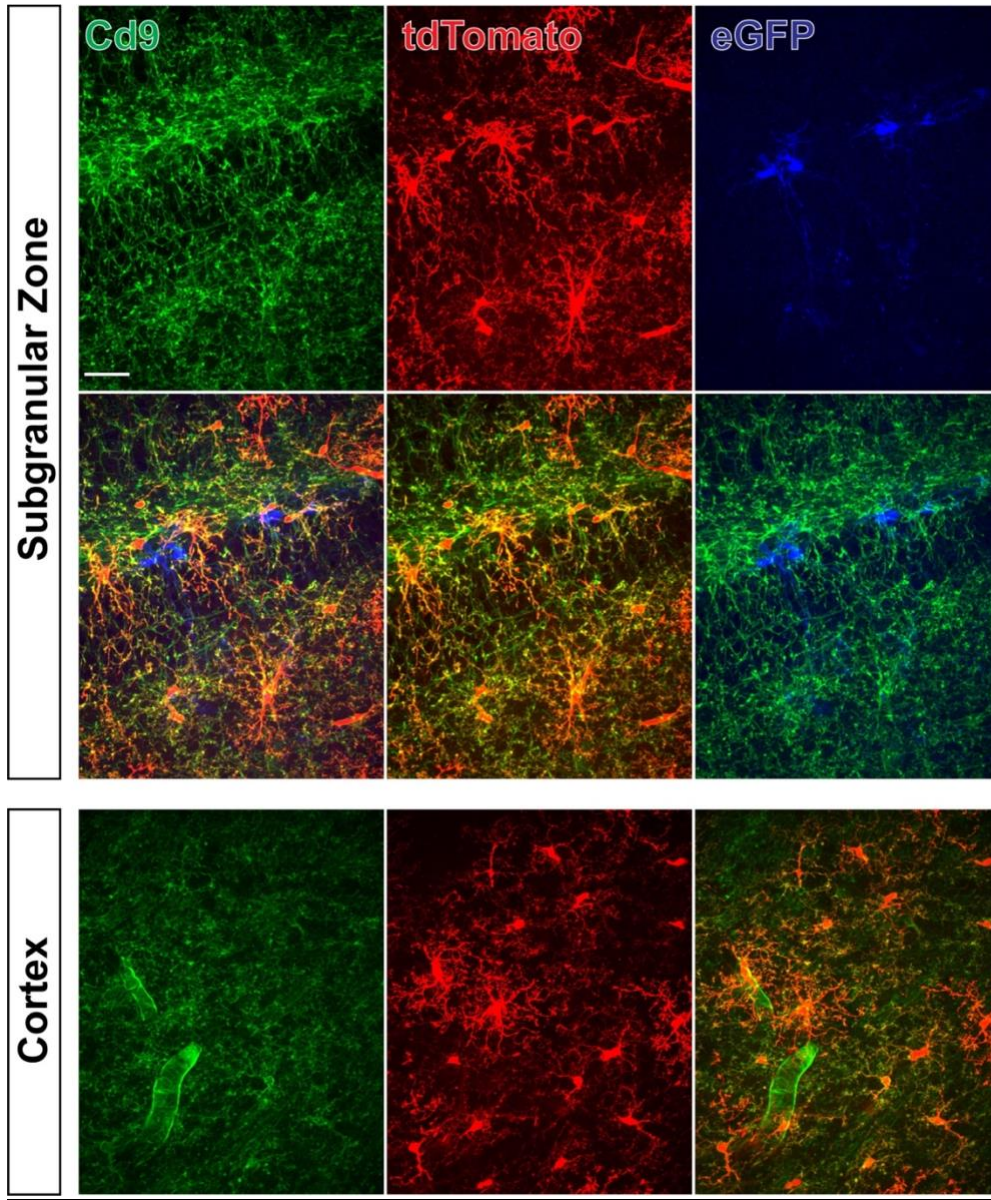

Supplement: S7 Fig — Cd9 immunoreactivity in dual reporter mice in individual channels (top) show expression of Cd9, tdTomato+ myeloid cells and eGFP+ neural progenitor cells. Merge images (middle) show colocalization of Cd9 in both tdTomato and GFP positive cells (left middle). Diffuse immunoreactivity in cortex (bottom) Scale = 10 μm. (PDF) [file pone.0296280.s007.pdf]

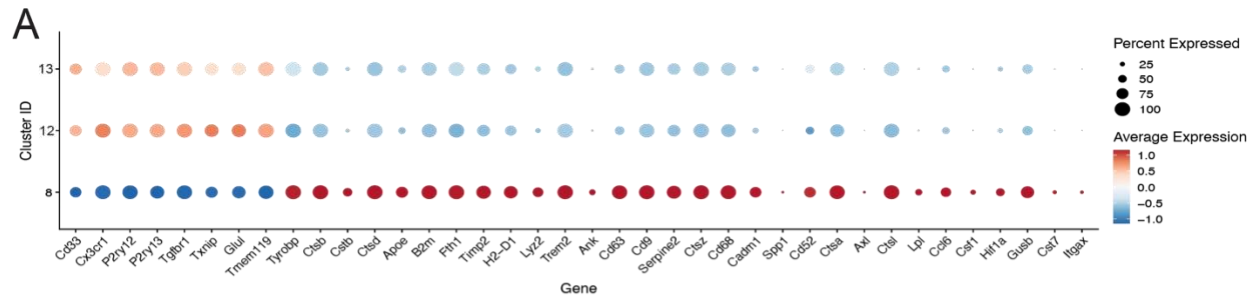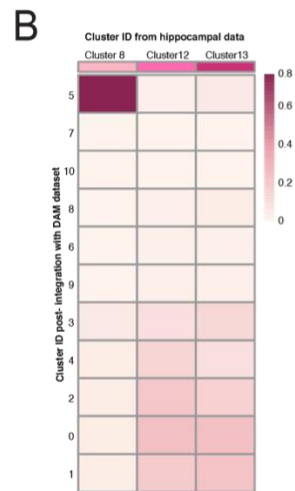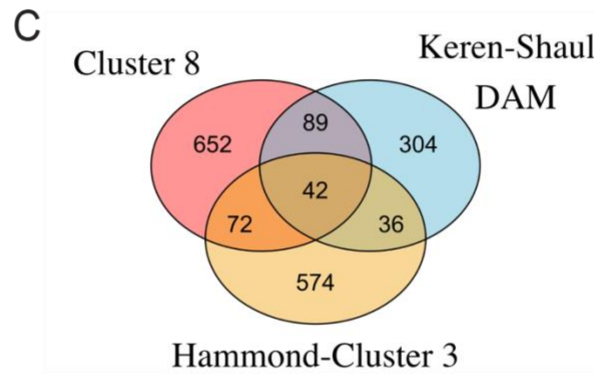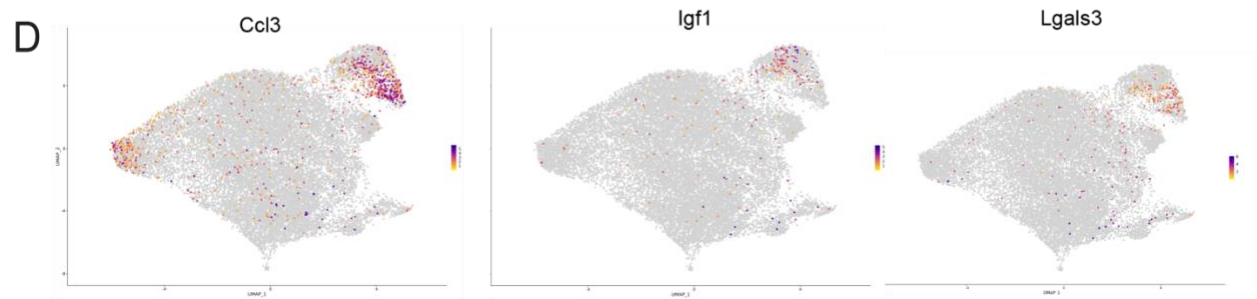

Supplement: S8 Fig — A. DAM marker gene expression levels in clusters 8, 12, and 13 of hippocampal myeloid cells. B. Correlation matrix showing split of Cross-dataset comparisons between cluster 8 microglia and reactive microglia. Plot showing relation between clusters 8,12,13 from this study and integrated dataset from Keren-Shaul et. al. 2017. Scale represents fraction of original cluster (from hippocampal myeloid dataset) represented in each integrated cluster from Fig 5E. C. Overlap of upregulated cluster 8, DAM, and early postnatal microglia (Hammond et. al. Cluster 3). D. Feature plots showing localization of Ccl3, Igf1, and Lgals3 as examples of genes enriched in reactive microglia from the intersection of genes featured in the venn diagram (part C of this figure). (PDF) [file pone.0296280.s008.pdf]
