# Supplementary material for: Distinct microglial transcriptomic signatures within the hippocampus
Source: PLoS One. 2024 Jan 5;19(1):e0296280. doi: 10.1371/journal.pone.0296280 (PMC10775894; doi:10.1371/journal.pone.0296280)
Supplement: S1 Table — Table displaying cells, mean transcript reads, mean number of unique genes, and the mean percentage of reads corresponding to mitochondrial genes in each sequencing sample. (DOCX) [file pone.0296280.s009.docx]

|  | Cells pre-filter | Mean reads | Mean genes | Mean % mito genes | Cells post-filter | Mean reads post filter | Mean genes post filter | Mean % mito genes |
| --- | --- | --- | --- | --- | --- | --- | --- | --- |
| Run 1 | 7255 | 4706 | 2014 | 3.47 | 6810 | 4885 | 2090 | 2.84 |
| Run 2 | 6895 | 3585 | 1621 | 2.49 | 5734 | 3935 | 1749 | 2.25 |
| Run 3 | 6226 | 3891 | 1792 | 3.09 | 5654 | 4109 | 1876 | 2.75 |
