# Supplementary material for: Distinct microglial transcriptomic signatures within the hippocampus
Source: PLoS One. 2024 Jan 5;19(1):e0296280. doi: 10.1371/journal.pone.0296280 (PMC10775894; doi:10.1371/journal.pone.0296280)
Supplement: S2 Table — (DOCX) [file pone.0296280.s010.docx]

|  | **GO.ID** | **Term** | **Annotated** | **Significant** | **Expected** | **Rank in classicFisher** | **classicFisher** | **classicKS** | **elimKS** |
| --- | --- | --- | --- | --- | --- | --- | --- | --- | --- |
| **1** | GO:0010033 | response to organic substance | 92 | 92 | 92 | 2 | 1 | 0.00037 | 0.00037 |
| **2** | GO:0045087 | innate immune response | 28 | 28 | 28 | 3 | 1 | 0.00041 | 0.00041 |
| **3** | GO:0008284 | positive regulation of cell population proliferation | 31 | 31 | 31 | 4 | 1 | 0.00047 | 0.00047 |
| **4** | GO:0044283 | small molecule biosynthetic process | 21 | 21 | 21 | 5 | 1 | 0.00049 | 0.00049 |
| **5** | GO:0001819 | positive regulation of cytokine production | 24 | 24 | 24 | 6 | 1 | 0.00050 | 0.00050 |
| **6** | GO:0060429 | epithelium development | 21 | 21 | 21 | 7 | 1 | 0.00068 | 0.00068 |
| **7** | GO:0030335 | positive regulation of cell migration | 27 | 27 | 27 | 8 | 1 | 5.7E-06 | 0.00071 |
| **8** | GO:0048732 | gland development | 14 | 14 | 14 | 9 | 1 | 0.00118 | 0.00118 |
| **9** | GO:0000902 | cell morphogenesis | 26 | 26 | 26 | 10 | 1 | 0.00148 | 0.00148 |
| **10** | GO:0031349 | positive regulation of defense response | 11 | 11 | 11 | 11 | 1 | 0.00157 | 0.00157 |
| **11** | GO:0050727 | regulation of inflammatory response | 14 | 14 | 14 | 12 | 1 | 0.00204 | 0.00204 |
| **12** | GO:0050920 | regulation of chemotaxis | 12 | 12 | 12 | 13 | 1 | 0.00208 | 0.00208 |
| **13** | GO:0002694 | regulation of leukocyte activation | 19 | 19 | 19 | 14 | 1 | 0.00215 | 0.00215 |
| **14** | GO:0002687 | positive regulation of leukocyte migration | 10 | 10 | 10 | 15 | 1 | 0.00239 | 0.00239 |
| **15** | GO:0007166 | cell surface receptor signaling pathway | 57 | 57 | 57 | 16 | 1 | 0.00243 | 0.00243 |
| **16** | GO:0030595 | leukocyte chemotaxis | 17 | 17 | 17 | 17 | 1 | 0.00262 | 0.00262 |
| **17** | GO:0016485 | protein processing | 11 | 11 | 11 | 18 | 1 | 0.00298 | 0.00298 |
| **18** | GO:0050866 | negative regulation of cell activation | 15 | 15 | 15 | 19 | 1 | 0.00317 | 0.00317 |
| **19** | GO:0006954 | inflammatory response | 30 | 30 | 30 | 20 | 1 | 2.7E-05 | 0.00350 |
| **20** | GO:0065008 | regulation of biological quality | 126 | 126 | 126 | 21 | 1 | 0.00184 | 0.00355 |
| **21** | GO:0043523 | regulation of neuron apoptotic process | 19 | 19 | 19 | 22 | 1 | 0.00379 | 0.00379 |
| **22** | GO:0009967 | positive regulation of signal transduction | 45 | 45 | 45 | 23 | 1 | 0.00385 | 0.00385 |
| **23** | GO:0009605 | response to external stimulus | 77 | 77 | 77 | 24 | 1 | 1.1E-05 | 0.00391 |
| **24** | GO:0022603 | regulation of anatomical structure morphogenesis | 36 | 36 | 36 | 25 | 1 | 0.00075 | 0.00445 |
| **25** | GO:0048729 | tissue morphogenesis | 12 | 12 | 12 | 26 | 1 | 0.00538 | 0.00538 |
| **26** | GO:2000026 | regulation of multicellular organismal development | 63 | 63 | 63 | 27 | 1 | 0.00205 | 0.00543 |
| **27** | GO:0035295 | tube development | 34 | 34 | 34 | 28 | 1 | 0.00109 | 0.00552 |
| **28** | GO:0043066 | negative regulation of apoptotic process | 39 | 39 | 39 | 29 | 1 | 0.00575 | 0.00575 |
| **29** | GO:0045861 | negative regulation of proteolysis | 10 | 10 | 10 | 30 | 1 | 0.00577 | 0.00577 |
| **30** | GO:0043065 | positive regulation of apoptotic process | 25 | 25 | 25 | 31 | 1 | 0.00587 | 0.00587 |
| **31** | GO:0097530 | granulocyte migration | 11 | 11 | 11 | 32 | 1 | 0.00612 | 0.00612 |
| **32** | GO:0045766 | positive regulation of angiogenesis | 12 | 12 | 12 | 33 | 1 | 0.00612 | 0.00612 |
| **33** | GO:0043085 | positive regulation of catalytic activity | 38 | 38 | 38 | 34 | 1 | 0.00118 | 0.00690 |
| **34** | GO:0032270 | positive regulation of cellular protein metabolic process | 48 | 48 | 48 | 35 | 1 | 0.00749 | 0.00749 |
| **35** | GO:0051235 | maintenance of location | 15 | 15 | 15 | 36 | 1 | 0.00765 | 0.00765 |
| **36** | GO:0006165 | nucleoside diphosphate phosphorylation | 10 | 10 | 10 | 37 | 1 | 0.00841 | 0.00841 |
| **37** | GO:0050867 | positive regulation of cell activation | 11 | 11 | 11 | 38 | 1 | 0.00864 | 0.00864 |
| **38** | GO:0002831 | regulation of response to biotic stimulus | 13 | 13 | 13 | 39 | 1 | 0.00882 | 0.00882 |
| **39** | GO:0051347 | positive regulation of transferase activity | 13 | 13 | 13 | 40 | 1 | 0.00897 | 0.00897 |
| **40** | GO:0042127 | regulation of cell population proliferation | 48 | 48 | 48 | 41 | 1 | 1.4E-05 | 0.00914 |
| **41** | GO:0006090 | pyruvate metabolic process | 10 | 10 | 10 | 42 | 1 | 0.00914 | 0.00914 |
| **42** | GO:0045595 | regulation of cell differentiation | 53 | 53 | 53 | 43 | 1 | 0.00919 | 0.00919 |
| **43** | GO:0022008 | neurogenesis | 50 | 50 | 50 | 44 | 1 | 0.00973 | 0.00973 |
| **44** | GO:0072503 | cellular divalent inorganic cation homeostasis | 16 | 16 | 16 | 45 | 1 | 0.01042 | 0.01042 |
| **45** | GO:0001503 | ossification | 12 | 12 | 12 | 46 | 1 | 0.01071 | 0.01071 |
| **46** | GO:0001906 | cell killing | 12 | 12 | 12 | 47 | 1 | 0.01135 | 0.01135 |
| **47** | GO:0002683 | negative regulation of immune system process | 19 | 19 | 19 | 48 | 1 | 0.01138 | 0.01138 |
| **48** | GO:0071621 | granulocyte chemotaxis | 10 | 10 | 10 | 49 | 1 | 0.01178 | 0.01178 |
| **49** | GO:1990266 | neutrophil migration | 10 | 10 | 10 | 50 | 1 | 0.01178 | 0.01178 |
| **50** | GO:0045597 | positive regulation of cell differentiation | 38 | 38 | 38 | 51 | 1 | 0.01242 | 0.01242 |
| **51** | GO:0051128 | regulation of cellular component organization | 73 | 73 | 73 | 52 | 1 | 0.01246 | 0.01246 |
| **52** | GO:0048646 | anatomical structure formation involved in morphogenesis | 31 | 31 | 31 | 53 | 1 | 0.00156 | 0.01250 |
| **53** | GO:0033674 | positive regulation of kinase activity | 11 | 11 | 11 | 54 | 1 | 0.01264 | 0.01264 |
| **54** | GO:0071310 | cellular response to organic substance | 76 | 76 | 76 | 55 | 1 | 0.01271 | 0.01271 |
| **55** | GO:0007399 | nervous system development | 66 | 66 | 66 | 56 | 1 | 0.00049 | 0.01299 |
| **56** | GO:0030030 | cell projection organization | 41 | 41 | 41 | 57 | 1 | 0.01318 | 0.01318 |
| **57** | GO:0002009 | morphogenesis of an epithelium | 10 | 10 | 10 | 58 | 1 | 0.01366 | 0.01366 |
| **58** | GO:0022610 | biological adhesion | 34 | 34 | 34 | 59 | 1 | 0.01372 | 0.01372 |
| **59** | GO:0051960 | regulation of nervous system development | 34 | 34 | 34 | 60 | 1 | 0.01372 | 0.01372 |
| **60** | GO:0009888 | tissue development | 43 | 43 | 43 | 61 | 1 | 6.8E-06 | 0.01461 |
| **61** | GO:0050678 | regulation of epithelial cell proliferation | 13 | 13 | 13 | 62 | 1 | 0.01473 | 0.01473 |
| **62** | GO:0051249 | regulation of lymphocyte activation | 13 | 13 | 13 | 63 | 1 | 0.01497 | 0.01497 |
| **63** | GO:0051094 | positive regulation of developmental process | 51 | 51 | 51 | 64 | 1 | 0.00374 | 0.01520 |
| **64** | GO:0002696 | positive regulation of leukocyte activation | 10 | 10 | 10 | 65 | 1 | 0.01600 | 0.01600 |
| **65** | GO:0031399 | regulation of protein modification process | 45 | 45 | 45 | 66 | 1 | 0.01652 | 0.01652 |
| **66** | GO:0048869 | cellular developmental process | 94 | 94 | 94 | 67 | 1 | 0.00032 | 0.01709 |
| **67** | GO:0051651 | maintenance of location in cell | 10 | 10 | 10 | 68 | 1 | 0.01740 | 0.01740 |
| **68** | GO:0002695 | negative regulation of leukocyte activation | 11 | 11 | 11 | 69 | 1 | 0.01750 | 0.01750 |
| **69** | GO:0007167 | enzyme linked receptor protein signaling pathway | 24 | 24 | 24 | 70 | 1 | 0.01762 | 0.01762 |
| **70** | GO:0042742 | defense response to bacterium | 10 | 10 | 10 | 71 | 1 | 0.01814 | 0.01814 |
| **71** | GO:0031175 | neuron projection development | 31 | 31 | 31 | 72 | 1 | 0.01845 | 0.01845 |
| **72** | GO:0002366 | leukocyte activation involved in immune response | 10 | 10 | 10 | 73 | 1 | 0.01847 | 0.01847 |
| **73** | GO:0042325 | regulation of phosphorylation | 44 | 44 | 44 | 74 | 1 | 0.01885 | 0.01885 |
| **74** | GO:0040012 | regulation of locomotion | 35 | 35 | 35 | 75 | 1 | 8.2E-06 | 0.01903 |
| **75** | GO:0010562 | positive regulation of phosphorus metabolic process | 34 | 34 | 34 | 76 | 1 | 0.01908 | 0.01908 |
| **76** | GO:0045937 | positive regulation of phosphate metabolic process | 34 | 34 | 34 | 77 | 1 | 0.01908 | 0.01908 |
| **77** | GO:0051962 | positive regulation of nervous system development | 23 | 23 | 23 | 78 | 1 | 0.01919 | 0.01919 |
| **78** | GO:0045859 | regulation of protein kinase activity | 17 | 17 | 17 | 79 | 1 | 0.01942 | 0.01942 |
| **79** | GO:0072507 | divalent inorganic cation homeostasis | 17 | 17 | 17 | 80 | 1 | 0.01942 | 0.01942 |
| **80** | GO:0030182 | neuron differentiation | 40 | 40 | 40 | 81 | 1 | 0.02021 | 0.02021 |
| **81** | GO:1901362 | organic cyclic compound biosynthetic process | 53 | 53 | 53 | 82 | 1 | 0.02023 | 0.02023 |
| **82** | GO:0050790 | regulation of catalytic activity | 57 | 57 | 57 | 83 | 1 | 0.00013 | 0.02059 |
| **83** | GO:0003013 | circulatory system process | 17 | 17 | 17 | 84 | 1 | 0.02080 | 0.02080 |
| **84** | GO:0008015 | blood circulation | 17 | 17 | 17 | 85 | 1 | 0.02080 | 0.02080 |
| **85** | GO:0030154 | cell differentiation | 91 | 91 | 91 | 86 | 1 | 0.00036 | 0.02083 |
| **86** | GO:0002521 | leukocyte differentiation | 18 | 18 | 18 | 87 | 1 | 0.02085 | 0.02085 |
| **87** | GO:0043549 | regulation of kinase activity | 18 | 18 | 18 | 88 | 1 | 0.02085 | 0.02085 |
| **88** | GO:0050808 | synapse organization | 13 | 13 | 13 | 89 | 1 | 0.02105 | 0.02105 |
| **89** | GO:0061564 | axon development | 13 | 13 | 13 | 90 | 1 | 0.02105 | 0.02105 |
| **90** | GO:0071345 | cellular response to cytokine stimulus | 30 | 30 | 30 | 91 | 1 | 0.02121 | 0.02121 |
| **91** | GO:0016052 | carbohydrate catabolic process | 14 | 14 | 14 | 92 | 1 | 0.02128 | 0.02128 |
| **92** | GO:0060627 | regulation of vesicle-mediated transport | 21 | 21 | 21 | 93 | 1 | 0.02137 | 0.02137 |
| **93** | GO:0009966 | regulation of signal transduction | 70 | 70 | 70 | 94 | 1 | 0.00031 | 0.02144 |
| **94** | GO:0007155 | cell adhesion | 33 | 33 | 33 | 95 | 1 | 0.02147 | 0.02147 |
| **95** | GO:0070887 | cellular response to chemical stimulus | 98 | 98 | 98 | 96 | 1 | 0.00264 | 0.02216 |
| **96** | GO:0031401 | positive regulation of protein modification process | 33 | 33 | 33 | 97 | 1 | 0.02219 | 0.02219 |
| **97** | GO:0048583 | regulation of response to stimulus | 96 | 96 | 96 | 98 | 1 | 3E-05 | 0.02314 |
| **98** | GO:0009617 | response to bacterium | 23 | 23 | 23 | 99 | 1 | 0.02316 | 0.02316 |
| **99** | GO:1901575 | organic substance catabolic process | 63 | 63 | 63 | 100 | 1 | 0.02410 | 0.02410 |
| **100** | GO:0002449 | lymphocyte mediated immunity | 10 | 10 | 10 | 101 | 1 | 0.02455 | 0.02455 |
| **101** | GO:0006874 | cellular calcium ion homeostasis | 15 | 15 | 15 | 102 | 1 | 0.02463 | 0.02463 |
| **102** | GO:0043524 | negative regulation of neuron apoptotic process | 15 | 15 | 15 | 103 | 1 | 0.02463 | 0.02463 |
| **103** | GO:0048468 | cell development | 57 | 57 | 57 | 104 | 1 | 0.02478 | 0.02478 |
| **104** | GO:0032102 | negative regulation of response to external stimulus | 13 | 13 | 13 | 105 | 1 | 0.02501 | 0.02501 |
| **105** | GO:0051480 | regulation of cytosolic calcium ion concentration | 13 | 13 | 13 | 106 | 1 | 0.02539 | 0.02539 |
| **106** | GO:0045785 | positive regulation of cell adhesion | 15 | 15 | 15 | 107 | 1 | 0.02569 | 0.02569 |
| **107** | GO:0000904 | cell morphogenesis involved in differentiation | 18 | 18 | 18 | 108 | 1 | 0.02613 | 0.02613 |
| **108** | GO:0051174 | regulation of phosphorus metabolic process | 50 | 50 | 50 | 109 | 1 | 0.02622 | 0.02622 |
| **109** | GO:0051047 | positive regulation of secretion | 20 | 20 | 20 | 110 | 1 | 0.02634 | 0.02634 |
| **110** | GO:0120036 | plasma membrane bounded cell projection organization | 37 | 37 | 37 | 111 | 1 | 0.02695 | 0.02695 |
| **111** | GO:0019438 | aromatic compound biosynthetic process | 50 | 50 | 50 | 112 | 1 | 0.02708 | 0.02708 |
| **112** | GO:0032879 | regulation of localization | 84 | 84 | 84 | 113 | 1 | 0.00357 | 0.02717 |
| **113** | GO:0006163 | purine nucleotide metabolic process | 16 | 16 | 16 | 114 | 1 | 0.02733 | 0.02733 |
| **114** | GO:0009150 | purine ribonucleotide metabolic process | 16 | 16 | 16 | 115 | 1 | 0.02733 | 0.02733 |
| **115** | GO:0009259 | ribonucleotide metabolic process | 16 | 16 | 16 | 116 | 1 | 0.02733 | 0.02733 |
| **116** | GO:0072521 | purine-containing compound metabolic process | 16 | 16 | 16 | 117 | 1 | 0.02733 | 0.02733 |
| **117** | GO:0002697 | regulation of immune effector process | 17 | 17 | 17 | 118 | 1 | 0.02763 | 0.02763 |
| **118** | GO:0006950 | response to stress | 113 | 113 | 113 | 119 | 1 | 0.00233 | 0.02847 |
| **119** | GO:0006875 | cellular metal ion homeostasis | 31 | 31 | 31 | 120 | 1 | 0.02903 | 0.02903 |
| **120** | GO:0034654 | nucleobase-containing compound biosynthetic process | 48 | 48 | 48 | 121 | 1 | 0.03007 | 0.03007 |
| **121** | GO:0051336 | regulation of hydrolase activity | 35 | 35 | 35 | 122 | 1 | 0.03025 | 0.03025 |
| **122** | GO:0007420 | brain development | 15 | 15 | 15 | 123 | 1 | 0.03047 | 0.03047 |
| **123** | GO:0048667 | cell morphogenesis involved in neuron differentiation | 15 | 15 | 15 | 124 | 1 | 0.03160 | 0.03160 |
| **124** | GO:0048858 | cell projection morphogenesis | 15 | 15 | 15 | 125 | 1 | 0.03160 | 0.03160 |
| **125** | GO:0045664 | regulation of neuron differentiation | 25 | 25 | 25 | 126 | 1 | 0.03246 | 0.03246 |
| **126** | GO:0048699 | generation of neurons | 46 | 46 | 46 | 127 | 1 | 0.03267 | 0.03267 |
| **127** | GO:0019725 | cellular homeostasis | 46 | 46 | 46 | 128 | 1 | 0.03312 | 0.03312 |
| **128** | GO:0042327 | positive regulation of phosphorylation | 32 | 32 | 32 | 129 | 1 | 0.03317 | 0.03317 |
| **129** | GO:0030155 | regulation of cell adhesion | 22 | 22 | 22 | 130 | 1 | 0.03324 | 0.03324 |
| **130** | GO:0043412 | macromolecule modification | 70 | 70 | 70 | 131 | 1 | 0.03326 | 0.03326 |
| **131** | GO:0001932 | regulation of protein phosphorylation | 39 | 39 | 39 | 132 | 1 | 0.03333 | 0.03333 |
| **132** | GO:0042063 | gliogenesis | 14 | 14 | 14 | 133 | 1 | 0.03340 | 0.03340 |
| **133** | GO:0019220 | regulation of phosphate metabolic process | 49 | 49 | 49 | 134 | 1 | 0.03370 | 0.03370 |
| **134** | GO:0050673 | epithelial cell proliferation | 15 | 15 | 15 | 135 | 1 | 0.03397 | 0.03397 |
| **135** | GO:0006464 | cellular protein modification process | 67 | 67 | 67 | 136 | 1 | 0.03408 | 0.03408 |
| **136** | GO:0036211 | protein modification process | 67 | 67 | 67 | 137 | 1 | 0.03408 | 0.03408 |
| **137** | GO:0045860 | positive regulation of protein kinase activity | 10 | 10 | 10 | 138 | 1 | 0.03582 | 0.03582 |
| **138** | GO:0050877 | nervous system process | 24 | 24 | 24 | 139 | 1 | 0.03647 | 0.03647 |
| **139** | GO:0030334 | regulation of cell migration | 33 | 33 | 33 | 140 | 1 | 2E-06 | 0.03675 |
| **140** | GO:0065009 | regulation of molecular function | 72 | 72 | 72 | 141 | 1 | 0.00046 | 0.03678 |
| **141** | GO:0060284 | regulation of cell development | 36 | 36 | 36 | 142 | 1 | 0.03678 | 0.03678 |
| **142** | GO:0048666 | neuron development | 34 | 34 | 34 | 143 | 1 | 0.03726 | 0.03726 |
| **143** | GO:0010720 | positive regulation of cell development | 26 | 26 | 26 | 144 | 1 | 0.03775 | 0.03775 |
| **144** | GO:0010646 | regulation of cell communication | 84 | 84 | 84 | 145 | 1 | 0.00068 | 0.03777 |
| **145** | GO:0023051 | regulation of signaling | 84 | 84 | 84 | 146 | 1 | 0.00068 | 0.03777 |
| **146** | GO:1902105 | regulation of leukocyte differentiation | 11 | 11 | 11 | 147 | 1 | 0.03789 | 0.03789 |
| **147** | GO:2000145 | regulation of cell motility | 35 | 35 | 35 | 148 | 1 | 8.2E-06 | 0.03955 |
| **148** | GO:0008285 | negative regulation of cell population proliferation | 17 | 17 | 17 | 149 | 1 | 0.03979 | 0.03979 |
| **149** | GO:0002263 | cell activation involved in immune response | 11 | 11 | 11 | 150 | 1 | 0.03987 | 0.03987 |
| **150** | GO:0002573 | myeloid leukocyte differentiation | 10 | 10 | 10 | 151 | 1 | 0.04051 | 0.04051 |
| **151** | GO:0030278 | regulation of ossification | 10 | 10 | 10 | 152 | 1 | 0.04116 | 0.04116 |
| **152** | GO:0003006 | developmental process involved in reproduction | 18 | 18 | 18 | 153 | 1 | 0.04134 | 0.04134 |
| **153** | GO:0010959 | regulation of metal ion transport | 15 | 15 | 15 | 154 | 1 | 0.04290 | 0.04290 |
| **154** | GO:1901615 | organic hydroxy compound metabolic process | 20 | 20 | 20 | 155 | 1 | 0.04301 | 0.04301 |
| **155** | GO:0055074 | calcium ion homeostasis | 16 | 16 | 16 | 156 | 1 | 0.04311 | 0.04311 |
| **156** | GO:0051641 | cellular localization | 72 | 72 | 72 | 157 | 1 | 0.04432 | 0.04432 |
| **157** | GO:0018130 | heterocycle biosynthetic process | 49 | 49 | 49 | 158 | 1 | 0.04479 | 0.04479 |
| **158** | GO:0009719 | response to endogenous stimulus | 35 | 35 | 35 | 159 | 1 | 0.04537 | 0.04537 |
| **159** | GO:0005975 | carbohydrate metabolic process | 23 | 23 | 23 | 160 | 1 | 0.04537 | 0.04537 |
| **160** | GO:0001501 | skeletal system development | 16 | 16 | 16 | 161 | 1 | 0.04609 | 0.04609 |
| **161** | GO:0050767 | regulation of neurogenesis | 31 | 31 | 31 | 162 | 1 | 0.04642 | 0.04642 |
| **162** | GO:0051050 | positive regulation of transport | 38 | 38 | 38 | 163 | 1 | 0.04837 | 0.04837 |
| **163** | GO:0005996 | monosaccharide metabolic process | 10 | 10 | 10 | 164 | 1 | 0.04991 | 0.04991 |
