# Supplementary material for: Distinct microglial transcriptomic signatures within the hippocampus
Source: PLoS One. 2024 Jan 5;19(1):e0296280. doi: 10.1371/journal.pone.0296280 (PMC10775894; doi:10.1371/journal.pone.0296280)
Supplement: Attachment — Submitted filename: Response to Reviewers_PLOS_6_2023.docx [file pone.0296280.s011.docx]

Response to Reviewers, original submission PONE-D-22-21184

Comments to the Author

Reviewer 1

1. Is the manuscript technically sound, and do the data support the conclusions?

The manuscript must describe a technically sound piece of scientific research with data that supports the conclusions. Experiments must have been conducted rigorously, with appropriate controls, replication, and sample sizes. The conclusions must be drawn appropriately based on the data presented. Partly
2. Has the statistical analysis been performed appropriately and rigorously? I Don't Know
3. Have the authors made all data underlying the findings in their manuscript fully available?

The PLOS Data policy requires authors to make all data underlying the findings described in their manuscript fully available without restriction, with rare exception (please refer to the Data Availability Statement in the manuscript PDF file). The data should be provided as part of the manuscript or its supporting information, or deposited to a public repository. For example, in addition to summary statistics, the data points behind means, medians and variance measures should be available. If there are restrictions on publicly sharing data—e.g. participant privacy or use of data from a third party—those must be specified. Yes
4. Is the manuscript presented in an intelligible fashion and written in standard English?

PLOS ONE does not copyedit accepted manuscripts, so the language in submitted articles must be clear, correct, and unambiguous. Any typographical or grammatical errors should be corrected at revision, so please note any specific errors here. Yes
5. Review Comments to the Author

Please use the space provided to explain your answers to the questions above. You may also include additional comments for the author, including concerns about dual publication, research ethics, or publication ethics. (Please upload your review as an attachment if it exceeds 20,000 characters) Chintamen et al., present a comprehensive profiling of myeloid lineage cells in the hippocampus by means of scRNA-sequencing. Noteworthy, they identify a microglia population, cluster 8, that expresses a gene signature with substantial overlap with Disease Associated Microglia. The authors claim that this population corresponds to microglial cells associated to progenitor cells in the SGZ.

Major

Despite the topic is of great interest, and it seems plausible that microglial cells associated to the neurogenic niche might have a distinctive gene profile, the data provided in the present manuscript is not enough to support such claims. The authors base most of their claim in immunofluorescent markers (CD68, CD9) that are not even quantified in the SGZ associated microglia.

*1. The article would benefit from adding quantifications to the histological analysis, especially on those markers that the authors relate to cluster 8 microglial population, such as CD68 and CD9. For instance, the authors claim that cluster 8 has increased cd68 levels compared with the rest of clusters, and they relate this with the increased CD68 signal in the microglia associated to the SGZ. However they fail to provide any quantification at the histological level to support this and they do not show images of the whole hippocampus to make clear that cd68 increases are restricted to the SGZ.
Perhaps the authors could compare the level of colocalization of these markers (cd68, cd9) between microglia cells in contact with the SGZ and microglial cells in other non neurogenic regions (hilus? cortex? ca1?). Moreover, since the authors claim that Tmem119 and Iba-1 are altered in the microglia from the SGZ (“these cells exhibit altered immunoreactivity to some common markers such as Tmem119 and Iba1”), appropriate quantification should be provided (colocalization, intensity, integrated density ?).*

We appreciate the thorough feedback from the reviewer. While protein-level differences would further corroborate and strengthen these findings (particularly in the context of spatial localization), we believe comparing immunofluorescent intensity in images is insufficient in determining enrichment of particular genes. This is particularly the case when comparing areas with varying cell density such as the cortex versus the SGZ. For the scope of this study, we focused on the transcriptomic atlas of the hippocampus and how that may explain potential discrepancies in conventionally employed marker genes. We agree that meaningful protein level quantitative analysis is important and would elevate the findings presented here, though they are simply outside the scope of what we are currently able to accomplish.

*2. Authors should temper down the text in general. Especially when relating the histological data with that of the scRNAseq. For instance (these are only examples, but the tempering should not be limited only to these):

“Transcriptomic analysis of the PUTATIVE subgranular zone cluster demonstrates unique expression profile”*

*“We note high specificity of Cd63 in the SGZ, FURTHER SUGGESTING that this transcriptomic cluster MIGHT BE specific to the neurogenic niche of the hippocampus (Supplemental Figure 7).”*

We appreciate these suggestions and agree that in some places we originally perhaps overemphasized particular findings. We have not only changed the text for the specific above comments in lines 495-497 to the suggested text above but have also newly annotated various other places throughout the manuscript where have tempered the language used.

*The data provided by the authors is not enough to state that cluster 8 corresponds unequivocally to SGZ-associated microglia. The text and the discussion should be toned down in this regard unless other approaches are included in the study (more IF markers specific for this putative population, ISH, RNAscope,…) that allow to make such claims. E.g. (but not limited to):

“Thus, we have characterized a key component of this neurogenic niche, a specialized subpopulation of microglia with a distinct transcriptomic signature correlating to function within this niche.”*

We agree with this assessment and have accordingly edited the manuscript. We have changed lines 580-581 to the following: “Thus, we have identified a subset of cells in the hippocampus with a distinct transcriptomic signature correlating to function within this niche.”

*“We identify a greater number of differentially expressed genes by subgranular zone microglia, and separate confounding results from CNS-associated macrophages. This approach allows us to computationally separate the immune population associated with the neurogenic niche of the hippocampal formation with great precision.”*

As suggested, we have reworked the text to temper these statements in lines 602-603 which now reads “This approach allows us to computationally separate different subsets of myeloid cells with great precision.”

*Please refrain from referring to cluster 8 as SGZ cluster in the text and the figures.
“We closely examined whether genes upregulated in the SGZ cluster overlapped with known activation profiles. We compared the SGZ transcriptome profile to that of Disease Associated Microglia (DAM) described in previous studies and found that microglia exclusively from the SGZ cluster with DAM (Figure 4E, Supplemental Figure 8) [8].”*

We have made these edits as suggested in in lines 530-534.

*3. Please provide more details in the material and methods section and the figure legends regarding the number of animals used in all experiments, especially in the histology ones. Authors should state in every figure legend the number of animals used for each of the panels.

There is no reference in the material and methods sections regarding the number of animals used, as well as the number of microglial cells reconstructed and analysed in each region for the sholl analysis. There is also no reference to the statistical test used to analyse this data. Please provide all the details.*

We have provided more information regarding the methods for Sholl analysis in lines 290-291 : “Cells for reconstruction were obtained a single section originating from one eight-old week male mouse.” Followed by further detail in lines 295-296: “These intersections were summed for each cell to obtain the total number of intersections and compared using an unpaired, Type 2 t-test.”

*Not clear whether the authors are pooling together hippocampi from the 4 sacrificed mice or only bilateral hippocampi from the same mouse. Authors mention in the results section that a total of 12 mice have been used for sc-RNAseq. The experimental design (number of animals, sex, repetitions, etc), should be clearly detailed in the material and methods.*

We have edited the text in lines 120-124 to reflect this information as follows:

“Each sequencing sample replicate comprised of four bilateral hippocampi from two female and two male, eight week old mice. We had a total of three replicates for single cell RNA-sequencing, resulting in cells analzyed from twelve bilateral hippocampi. Our data set consists of cells from hippocampi originating from a total of six male mice and six female mice.”

*4. The authors should also give a brief explanation of cluster 11 as they do for the other clusters that do not meet the threshold.

“Clusters 4,5,6, and 11 did not meet the threshold for differential gene expression for gene set enrichment analysis.”*

This has been addressed- we have included a brief explanation of cluster 11 in lines 390-391 as described below:“Lastly, Cluster 11 shows enrichment of *Pmepa1* has been identified in TGF-ß signaling.”We have also included a reference to Brunschwig et al 2003 who found a role of Pmepa1 in regulating TGF-B signaling.

5. *“We next determined whether there were sex-specific differences in the transcriptome profiles of cells originating from male versus female samples.”

I would like to know the advantages to do 3 technical replicates (each one with 2 females and 2 males pooled) instead of performing separate experiments with only males and only females to determine sex-specific differences. I would also like to know how reliable is Xist expression to identify female-derived cells and how often is it used in the scRNAseq field. It would be nice if the authors could refer in the text to other articles using this same methodology to determine sex-specific differences in scRNAseq.*

These were done to eliminate batch-dependent effects. As many mouse studies not explicitly focused on sex-specific effects tend to exclude samples derived from female mice, this approach is not commonly used. However, there is validation for it and Gaublomme et al 2019 observed 98% agreement in demultiplexed cells labelled with antibodies conjugated to hashtag oligonucleotides and cells classified using Xist expression. In a recently published study from Anderson and others, 2023; the authors also pooled male and female samples as a means to pool samples originating from one male and female for demultiplexing.

*6. Less ramified microglial cells are usually considered as somehow activated or at least non-homeostatic, however that does not mean that they are phagocytic. There are many activation states, and not all of them are related with phagocytosis. In addition, microglial activation is usually accompanied of increased soma area and thicker ramifications. Have the authors considered measuring other morphological parameters?*

We attempted to measure these morphological features using the tdTomato mice. However, the high density of cells in the SGZ with multiple overlapping processes make it extremely challenging to resolve signal from adjacent cells for other forms of morphometric analysis.

*“We find that cells with their cell bodies located in the sub granular zone are less ramified than cells in the cortex (Figure 4C), which is consistent with a phagocytic phenotype [45, 46].”*

7. *According to the authors, there is high density of microglia around the progenitors of the SGZ, the neurogenic area of the hippocampus. This is at odds with the data from the SVZ. The authors observe lower density of microglia in the GL but not in the SGZ.
“We also observed decreased immunoreactivity of Iba1+ cell processes in the granular layer of the dentate gyrus, similar to reports in the subventricular zone where this other brain neurogenic region also demonstrates lower Iba1 expression.”*

Our studies and others have implicated heterogeneous marker gene expression within subpopulations of microglia. Xavier and others have shown that roughly half of cells that are CX3CR1-EGFP+ in the SVZ are not captured by Iba1 immunostaining.

8. *There is intense cd68 immunostaining in regions of the hippocampus other than the SGZ, such as the hilus, and also below the DG infrapyramidal blade (upper part of image 2C; I think the hippocampus might be upside-down). Please comment on that and explain why part of this signal does not co-localize with iba-1. Is it unspecific?
Have the authors checked whether this same histological profile (e.g. increased CD68 puncta) also occurs in the microglia associated to other neurogenic regions such as de VZ/SVZ?*

Non-myeloid cells also express Cd68. Image 2 contains tdTomato labeling of myeloid cells under Cx3Cr1 Cre recombination. The cells expressing high levels of Cd68 surrounding the blood vessels may be endothelial cells which are known to express Cd68 (Gottfried et. al. 2018). While this section of the hippocampus appears upside down relative to ventral sections, it is in fact slightly dorsal relative to the other section featured in the figure. In dorsal coronal sections the murine dentate gyrus seemingly inverts over the dorsal-ventral axis. Xavier et al 2015 observed an increase in CD68+ cells in the olfactory bulb. We have cited this reference in line 414.While neural stem/progenitor cells do not originate in the olfactory bulb, they eventually migrate to this region where they are engulfed in a manner similar to the engulfment of immature neurons in the neurogenic pool of the dentate gyrus.


Minor

9. *Duplicities and misspells

Correct Mice
“Seven-week old dual reporter mice mice were injected with Tamoxifen (100mg/kg) once a day for four consecutive days.”

Niche seems to be misspelled
“further suggesting that this cluster of cells correlates to microglia spatially
aligned to the neurogenic nice”

Sex-specific is misspelled in the Figure legend of Supl. Fig3.

The last “and” should be removed.

“However, in our dataset these cells express microglia marker genes and
no border macrophage genes such as Mrc1, Ms4a7, and Pf4 and were detected.”*

We thank the reviewer for catching these errors and have addressed these errors.

*10. Missing and incorrect references

Reference 40, does not mention that CD68 is found in the SGZ. Please revise.*

This reference was initially to highlight the role of CD68 in many cellular processes often but not exclusively found in macrophages and other myeloid cells. As mentioned above have clarified this statement in lines 437-439. “Cd68, (macrosialin) is found on the surface of lysosomes and increased Cd68 expression is detected in regions with neuronal death such as the cerebellum and olfactory bulb.”

*Who are the others? Please provide the appropriate reference.
“We and others have noted deviation in SGZ microglia from the tiled distribution of microglia found elsewhere (such as the cortex) (Figure 4A, 4B) in the homeostatic brain.”*

We have expanded this to clarify that microglia in the cortex are tiled while other regions with high neuronal density do not have such tiling and cited these statements accordingly. These changes are reflected in lines 514-519:

“While the distribution of microglia in the resting cortex is characterized as tiled with microglial branches from adjacent cells maintaining distinct, non-overlapping territories, some regions of the brain do not have this patterning, particularly in regions with high neuronal densities [1, 2]. We noted deviation in SGZ microglia from this tiled distribution compared to cortical microglia (Figure 5A, 5B) in the homeostatic brain.”

*Please provide reference regarding iba-1 in the SVZ.
Similar to microglia in the adult subventricular zone, these cells exhibit altered immunoreactivity to some common markers such as Tmem119 and Iba1.*

We have added a reference to Xavier et. al. 2015 in lines 591-593.

*Please provide references for the first paragraph of the discussion.
“As the involvement of immune cells in CNS development and health are examined in greater depth, the role of innate immune cells reveals diverse function depending on cell type and context. Different cell types are tuned to recognize and respond to specific cues. Some populations present in the brain express different sets of genes which may explain mechanisms involving aberrant immune activation and inflammation leading to neurodegenerative diseases coupled with cognitive decline. Immune cells are undoubtedly an important layer of defense for the central nervous system, while dysregulation of the neuroimmune axis can disrupt healthy neural and cognitive functions.”*

To support these statements we have cited a number of papers and reviews to support these statements. I have listed them below:

As the involvement of immune cells in CNS development and health are examined in greater depth, the role of innate immune cells reveals diverse function depending on cell type and context [17,36,51]

17.       Chintamen S, Imessadouene F, Kernie SG. Immune Regulation of Adult Neurogenic Niches in Health and Disease. Front Cell Neurosci. 2020;14:571071. Epub 2021/02/09. doi: 10.3389/fncel.2020.571071. PubMed PMID: 33551746; PubMed Central PMCID: PMC7855589.

36.    Li Q, Barres BA. Microglia and macrophages in brain homeostasis and disease. Nat Rev Immunol. 2018;18(4):225-42. Epub 2017/11/21. doi: 10.1038/nri.2017.125. PubMed PMID: 29151590.

51. Butovsky O, Weiner HL. Microglial signatures and their role in health and disease. Nat Rev Neurosci. 2018;19(10):622-35. Epub 2018/09/13. doi: 10.1038/s41583-018-0057-5. PubMed PMID: 30206328; PubMed Central PMCID: PMC7255106.

Different cell types are tuned to recognize and respond to specific cues[37, 52, 53].

37.       Prinz M, Masuda T, Wheeler MA, Quintana FJ. Microglia and Central Nervous System-Associated Macrophages-From Origin to Disease Modulation. Annu Rev Immunol. 2021;39:251-77. Epub 2021/02/09. doi: 10.1146/annurev-immunol-093019-110159. PubMed PMID: 33556248; PubMed Central PMCID: PMC8085109.

52.       Ransohoff RM, Perry VH. Microglial physiology: unique stimuli, specialized responses. Annu Rev Immunol. 2009;27:119-45. Epub 2009/03/24. doi: 10.1146/annurev.immunol.021908.132528. PubMed PMID: 19302036.

53.       Kierdorf K, Masuda T, Jordao MJC, Prinz M. Macrophages at CNS interfaces: ontogeny and function in health and disease. Nat Rev Neurosci. 2019;20(9):547-62. Epub 2019/07/31.

Some populations present in the brain express different sets of genes which may explain mechanisms involving aberrant immune activation and inflammation leading to neurodegenerative diseases coupled with cognitive decline [8, 54-56]

8.Keren-Shaul H, Spinrad A, Weiner A, Matcovitch-Natan O, Dvir-Szternfeld R, Ulland TK, et al. A Unique Microglia Type Associated with Restricting Development of Alzheimer's Disease. Cell. 2017;169(7):1276-90 e17. Epub 2017/06/13. doi: 10.1016/j.cell.2017.05.018. PubMed PMID: 28602351.

54.       Safaiyan S, Besson-Girard S, Kaya T, Cantuti-Castelvetri L, Liu L, Ji H, et al. White matter aging drives microglial diversity. Neuron. 2021;109(7):1100-17 e10. Epub 2021/02/20. doi: 10.1016/j.neuron.2021.01.027. PubMed PMID: 33606969.

55.       Mathys H, Adaikkan C, Gao F, Young JZ, Manet E, Hemberg M, et al. Temporal Tracking of Microglia Activation in Neurodegeneration at Single-Cell Resolution. Cell Rep. 2017;21(2):366-80. Epub 2017/10/12. doi: 10.1016/j.celrep.2017.09.039. PubMed PMID: 29020624; PubMed Central PMCID: PMC5642107.

56.       Jordao MJC, Sankowski R, Brendecke SM, Sagar, Locatelli G, Tai YH, et al. Single-cell profiling identifies myeloid cell subsets with distinct fates during neuroinflammation. Science. 2019;363(6425). Epub 2019/01/27. doi: 10.1126/science.aat7554. PubMed PMID: 30679343.

Immune cells are undoubtedly an important layer of defense for the central nervous system, while dysregulation of the neuroimmune axis can disrupt healthy neural and cognitive functions[57-60].

57.  Glass CK, Saijo K, Winner B, Marchetto MC, Gage FH. Mechanisms underlying inflammation in neurodegeneration. Cell. 2010;140(6):918-34. Epub 2010/03/23. doi: 10.1016/j.cell.2010.02.016. PubMed PMID: 20303880; PubMed Central PMCID: PMC2873093.

58.   Liddelow SA, Guttenplan KA, Clarke LE, Bennett FC, Bohlen CJ, Schirmer L, et al. Neurotoxic reactive astrocytes are induced by activated microglia. Nature. 2017;541(7638):481-7. Epub 2017/01/19. doi: 10.1038/nature21029. PubMed PMID: 28099414; PubMed Central PMCID: PMC5404890.

59.   Cribbs DH, Berchtold NC, Perreau V, Coleman PD, Rogers J, Tenner AJ, et al. Extensive innate immune gene activation accompanies brain aging, increasing vulnerability to cognitive decline and neurodegeneration: a microarray study. J Neuroinflammation. 2012;9:179. Epub 2012/07/25. doi: 10.1186/1742-2094-9-179. PubMed PMID: 22824372; PubMed Central PMCID: PMC3419089.

60.   Villeda SA, Luo J, Mosher KI, Zou B, Britschgi M, Bieri G, et al. The ageing systemic milieu negatively regulates neurogenesis and cognitive function. Nature. 2011;477(7362):90-4. Epub 2011/09/03. doi: 10.1038/nature10357. PubMed PMID: 21886162; PubMed Central PMCID: PMC3170097.

This makes characterizing the different players in the immune system key to uncovering pathological development and recovery in neurological diseases[18, 61, 62]

18.       Willis EF, MacDonald KPA, Nguyen QH, Garrido AL, Gillespie ER, Harley SBR, et al. Repopulating Microglia Promote Brain Repair in an IL-6-Dependent Manner. Cell. 2020;180(5):833-46 e16. Epub 2020/03/07. doi: 10.1016/j.cell.2020.02.013. PubMed PMID: 32142677.

61.       Elmore MRP, Hohsfield LA, Kramar EA, Soreq L, Lee RJ, Pham ST, et al. Replacement of microglia in the aged brain reverses cognitive, synaptic, and neuronal deficits in mice. Aging Cell. 2018;17(6):e12832. Epub 2018/10/03. doi: 10.1111/acel.12832. PubMed PMID: 30276955; PubMed Central PMCID: PMC6260908.

62.       Rogers JT, Morganti JM, Bachstetter AD, Hudson CE, Peters MM, Grimmig BA, et al. CX3CR1 deficiency leads to impairment of hippocampal cognitive function and synaptic plasticity. J Neurosci. 2011;31(45):16241-50. Epub 2011/11/11. doi: 10.1523/JNEUROSCI.3667-11.2011. PubMed PMID: 22072675; PubMed Central PMCID: PMC3236509.

11. Scale bars and figures

*Suppl. Fig 2
In order to assess better the quality control metrics between the 3 technical replicates, the authors should consider to use the same Y axis range in the violin plots of the 3 experiments.*

We have addressed this by applying a uniform Y-axis across the 3 replicates for each of the graphs in supplemental Figure 2.

*Suppl. Fig 3
The figure legend makes reference to D “Breakdown of genes differentially expressed genes (highlighting sex-spcific clustering) in homeostatic clusters.”, but there is no D in the actual figure.*

We have edited the supplemental figure to omit Supplemental Figure 3D which was moved to Supplemental Figure 5

*Fig.2, 3
Please revise the scale bars. Some of them are not present in the image.*

Figure 2 A-D are single channels and an overlay of the same field of view. Therefore, the scale in 2A represents the scale for 2B,2C, and 2D. Furthermore 2 G is from the inset. We have included a scale bar for this image. We have moved images formerly in Figure 3 to Figure 4- the scale is the same for images from both subpanels but to illustrate this more clearly, we have added an additional scale bar in Figure 4B.

*The figures and supplementary figures should be labelled in order of appearance. For instance, Sup. Fig. 7 is mentioned in the text before sup. Fig. 6.*

We have relabeled these figures and edited the text in line 468 for Supplemental figure 6 and lines 453-454 for the first reference to Supplemental figure 7.

*Figure legend 3.
arrowheads in E are not exclusively in the SGZ. Please correct.
“Decreased Tmem119 immunoreactivity in SGZ (arrow in middle panel) and sparse colocalization with Iba1 in SGZ indicated by arrowheads (right panel).”*

We have resolved these by limiting arrowheads to the SGZ. These figures have been relabeled as Figure 4 and the revised positions of the arrows and their descriptions can be found in Figure 4 and its figure legend, respectively.

6. PLOS authors have the option to publish the peer review history of their article (what does this mean?). If published, this will include your full peer review and any attached files.

If you choose “no”, your identity will remain anonymous but your review may still be made public.

Do you want your identity to be public for this peer review? For information about this choice, including consent withdrawal, please see our Privacy Policy. No
Confidential to Editor

1. Do you have any potential or perceived competing interests that may influence your review? Please review our Competing Interests policy and declare any potential interests that you feel the Editor should be aware of when considering your review. If you have no competing interests, please write "I have no competing interests." I have no competing interests that might influence my review.
2. Did you receive any assistance in preparing this review (e.g. from a post-doc or graduate student)? If yes, please include their name below. No
3. If accepted, do you think this submission should be highlighted on the PLOS ONE website? PLOS ONE does not evaluate manuscripts based on perceived significance or readership. We aim to provide tools for readers to filter and evaluate our publications. (optional) No
Do you want to get recognition for this review on Publons?
If you opt in, your Publons profile will automatically be updated to show a verified record of this review in full compliance with the journal’s review policy. If you don’t have a Publons profile, you will be prompted to create a free account.

No


Reviewer 2

1. Is the manuscript technically sound, and do the data support the conclusions?

The manuscript must describe a technically sound piece of scientific research with data that supports the conclusions. Experiments must have been conducted rigorously, with appropriate controls, replication, and sample sizes. The conclusions must be drawn appropriately based on the data presented. Partly
2. Has the statistical analysis been performed appropriately and rigorously? Yes
3. Have the authors made all data underlying the findings in their manuscript fully available?

The PLOS Data policy requires authors to make all data underlying the findings described in their manuscript fully available without restriction, with rare exception (please refer to the Data Availability Statement in the manuscript PDF file). The data should be provided as part of the manuscript or its supporting information, or deposited to a public repository. For example, in addition to summary statistics, the data points behind means, medians and variance measures should be available. If there are restrictions on publicly sharing data—e.g. participant privacy or use of data from a third party—those must be specified. Yes
4. Is the manuscript presented in an intelligible fashion and written in standard English?

PLOS ONE does not copyedit accepted manuscripts, so the language in submitted articles must be clear, correct, and unambiguous. Any typographical or grammatical errors should be corrected at revision, so please note any specific errors here. Yes
5. Review Comments to the Author

Please use the space provided to explain your answers to the questions above. You may also include additional comments for the author, including concerns about dual publication, research ethics, or publication ethics. (Please upload your review as an attachment if it exceeds 20,000 characters) Chintamen et al., explore at single cell level, the transcriptional profiles of acutely isolated myeloid lineage cells in the hippocampus. The authors reveal heterogeneity of transcriptional microglia types including a population (cluster 8) showing reduced expression of gene markers of homeostatic microglia and increased levels of transcripts functionally related to phagocytosis. Based in histological analysis of selected markers the authors claim that this population correspond to specialized microglia residing in the neurogenic niche in the subgranular zone (SGZ) of the hippocampal dentate gyrus. While in principle, this is a timely and interesting study, there are also a number of issues in the data shown, the conclusions drawn and the overall presentation of the manuscript that undermine the paper.

Major:

*1.- The main concern of the study is the claim that cluster 8 corresponds to microglia specialized in the neurogenic niche of the hippocampus. For this reviewer, the data presented are not convincing to support this conclusion.

The authors provide compelling evidence that cluster 8 expresses a transcriptional profile consistent with phagocytic activity (Figure 3A, B). This population shows substantial overlap with DAM microglia that cluster around amyloid plaques in the brain of Alzheimer's disease models (Keren-Shaul et al., 2017) and may be involved in plaque clearance (Huang et al., 2021) (Figure 4). On the other hand, morphological analysis shows that microglia in the SGZ display reduced levels of branching compared to microglia resident in the cortex, a feature associated with phagocytic activities (Kettenmann, 2007). This is consistent with the high rate of apoptosis of newborn cells in the adult SGZ and the role of microglia in eliminating SGZ cells undergoing apoptosis (Sierra et al., 2010). Therefore, it is reasonable to hypothesize that cluster 8 corresponds to microglia in the hippocampal neurogenic niche. However, the evidence presented to validate this hypothesis is very weak.

First, selected genes enriched in cluster 8, such as Cd68 (Figure 2), Cd9 (Figure 4, Suppl. Fig. 6, 7) and Cd63 (Suppl. Fig. 7), are also expressed at good levels in all other clusters (see Table 1 for percentage values). In addition, Cd9 is expressed in resident populations such as GFAP-positive quiescent neural stem cells (Marques-Torrejon et al., 2021). Cd63 is also present in neural stem cells of the adult neurogenic niche (Morton et al., 2018). The presence of these confounding factors compromises the conclusions of the histological analyses and also, that of the Allen Brain Atlas ISH data for individual probes. Therefore, additional spatial information is critical to assign cluster 8 to the microglia populating the SGZ. This could include multiplex RNAscope of markers selected for cluster 8 such as Cd83, C3ar1, Cadm1, which show low levels of detection in cells annotated to other clusters (see pct values in Table 1), showing colocalization with bona fide microglia markers (Hexb, Tmem119, Siglech). However, strategies based on selected candidates are always limited and new high-throughput methods with spatial resolution, such as spatial transcriptomics or proteomics, seem better suited to elucidate the heterogeneity of microglia populations within the hippocampal region.*

The reviewer brings up a compelling point around the specificity of cluster 8 to the SGZ. We agree that some of these markers are not exclusive to the cluster 8 and in fact not exclusive to microglia. However, we believe that the combination of different marker genes is unique to this subset of cells. Immune cells from other tissues are often separated using combinatorial panels of marker genes and similarly, deeper studies of microglia with varied phenotypes may also require the use of a combination of marker genes.

While spatial transcriptomics would be preferable, at the time of this study commercially available platforms for spatial transcriptomics were limited to low spatial resolution (roughly 50 µm) with less coverage (large gaps between sequencing spots). For areas with substantially high cell density such as the dentate gyrus and subgranular zone, this results in reads captured from multiple cells potentially obscuring differences between microglia and resulting in low coverage of regional microglia.

*Second, the images shown in Figure 3D, E which are critical for assigning cluster 8 cells to the microglia populating the SGZ, are of poor quality and should be improved.*

In order to make the images formerly in Figures 3D and 3E more visible, we have provided them as a separate figure (Figure 4) with enhanced resolution that allow the reader to more clearly appreciate the expression (or lack thereof) for markers specific to this cluster.

*3.- Methods section. Normalization and integration. The manuscript indicates that filtering of low-quality cells was performed by setting a threshold of “20% of total UMIs for mitochondrial genes”. However, this filtering criterion seems to differ from that shown in Suppl. Fig. 2, which seems more appropriate (e.g., Osorio and Cai. 2021). Please clarify this point.*

In order to filter out low quality cells, we filter out cells expressing too few genes or have too few transcript reads. We have clarified these metrics in lines 194-198. Notably, there is only a minor difference between the number of cells filtered out when using 10% as the filter (18.169 cells pass) versus 20% (18,198 cells pass the threshold).

*4.- Methods. Doublets are a key confounder factor in scRNA-seq data analysis and should be identified and filtered out. There are methods such as Scrublet, DoulbetFinder and others that can help identify and eliminate doublets (e.g. Miles et al., 2021).*

Since DoubletFinder relies on a predicted or expected incidence of doublets, we were not able to obtain an accurate metric based on empirical data for this cell type. However, we ran Doubletfinder based on commonly used values for PBMCs which may behave similar to microglia. With this strategy applied, we found only a handful of cells classified as doublets spread across clusters without enrichment in any one cluster.

*5.- Methods section. Dimensionality reduction and clustering. The rationale for setting the resolution parameter to 0.5 and 20 PCs is not clear. Microglia populations such as homeostatic (cluster 12,13,14), proliferative (cluster 1), interferon-related (cluster 3), DAMs (cluster 8,10...), have been consistently revealed through mouse and human studies, and some populations have also been identified in situ with spatial transcriptomics. However, although the clusters found with these parameters fit well with the UMAP plot shown in Fig.1D, the total number of clusters identified seems very high and many of them may not be biologically relevant. Indeed, many clusters, including clusters 4,5,6 and 11, do not show differentially expressed genes, which may indicate that the cells are over clustered (Table 1, Suppl. Fig. 3C, 4). Analysis based on silhouette scores per cluster can help in choosing the resolution parameter (e.g. Patterson-Cross et al., 2021). In addition, high gene detection and stringent filtering of the data to only include true cells that are of high quality can positively impact on clustering and cell type classification. The authors should extend the filtering to remove undesirable cells beyond the mito genes. High quality cells should be selected based also on the number of UMIs and number of detected genes. Finally, using scores to aggregate the expression levels of a given gene signature can help to filter out low-quality or stressed cells (e.g., cluster 9) (Marsh et al., 2022).*

For the purposes of this manuscript, we sought to maintain resolution between clusters. This is illustrated in supplemental Figure 3C in which different clusters have different levels of key marker genes, particularly Aif1 and CD68. We are now specifying our filtering criteria cells based on UMIs and number of detected genes.

Additionally, we chose resolution based on when cell cluster identity corresponded with UMAP localization. For instance, at lower resolutions, cells located distal to one of another would be clustered together. Notably, at lower resolutions, cluster 8 remains distinct.

We also noted that we used SCTransform and in this we clarified that we regressed out percent mitochondrial genes in lines 202-203.

6.- Please clarify the selection criteria for the genes shown in the dot plot in Fig.1E, in the density map in Fig.1F and in the dot plot in Suppl. Fig.3.

We incorporated this feedback and added detail to clarify how marker genes were used to determine and highlight the purity of the myeloid cell population sequenced in lines 341-356. Below is the detailed explanation provided in the main text:

Cells from all clusters expressed common marker genes such as *Cx3cr1*, *Cd11b* (*Itgam*), and *Cd45* (*Ptprc*) that are known to be enriched in myeloid lineage cells such microglia and other macrophages (Figure 1E). We next plotted relative transcript levels of marker genes found broadly in myeloid lineage cells (Iba1 aka Aif1), monocyte (ccr2), , those specific to microglia (*Tmem119, P2ry12, and Hexb)* Supplemental Figure 3C). Some clusters had small fractions of cells with negligible levels of marker genes from other immune cells such as T cells (*Cd4*, *Cd8a*), B cells (*Cd19* , *Cd80*), neurons (*Rbfox* aka NeuN), NG2 glia (*Cspg4*), neuronal stem cells (*Nes*, *Gfap*), astrocytes (*Gfap*), and oligodendrocytes (*Mbp* and *Olig1*) but in large part these transcripts are not detected at appreciable levels or in most cells (Figure 1E). These may reflect material from adjacent cells in close association with isolated microglia or alternatively transient expression of these genes as is the case in newly formed microglia with *Nestin* expression [3]. In addition, we examined in further detail marker genes to resolve types of myeloid lineage cells. We noted relatively consistent levels of microglial specific genes such as *Tmem119, P2ry12,* and *Hexb* (Supplemental Figures 3C & 4) across most clusters with the exception of two clusters which we describe in greater detail below.

7.- *Cd68 is expressed in all clusters and its transcript levels are increased in cluster 8. Quantification of histological analysis shown in Figure 2 could reveal differences in Cd68 protein levels between microglia in SGZ and other brain areas such as the ML or CTX.*

We agree with these statements, however we believe the methods required to resolve protein-level differences in this region may be outside of the scope of this study.

Minor:
*1.- Cluster 2 is assigned to non-microglia macrophage population based on high levels of Cd45 (Ptprc). Some cells in this cluster show high levels of Mbp. The same is true for cluster 4 which shows many Gfap-positive cells. In addition, the presence of transcripts coding for Rbfox3 (Nes) is found in some cells in cluster 8. Please, discuss these findings.*

This may reflect engulfment of matter from these cells. In the case of Nestin, this particular gene has been shown to be expressed in microglia under specific circumstances. Elmore and others noted its expression during early repopulation of microglia post-ablation via pharmacological inhibition of CSF1R. I have included this in the main text in lines 346-352 as follows:

“Some clusters had small fractions of cells with negligible levels of marker genes from other immune cells such as T cells (*Cd4*, *Cd8a*), B cells (*Cd19* , *Cd80*), neurons (*Rbfox* aka NeuN), NG2 glia (*Cspg4*), neuronal stem cells (*Nes*, *Gfap*), astrocytes (*Gfap*), and oligodendrocytes (*Mbp* and *Olig1*) but in large part these transcripts are not detected at appreciable levels or in most cells (Figure 1E). These may reflect material from adjacent cells in close association with isolated microglia or alternatively transient expression of these genes as is the case in newly formed microglia with *Nestin* expression [3].”

2.- Methods section. Normalization and integration. The integration method should be better described here (for example, indicate here the use of CCA to integrate the libraries).

We have included more detail surrounding normalization steps in lines 202-217, specifically the specification where we regressed out percent mitochondrial genes as a factor that would influence normalization and downstream clustering. We listed CCA as the method of integration in line 213.

*3.- High density of microglia in the SGZ. This is an interesting observation that deserves to be analyzed and quantified in greater depth (for example, taking the cortex as a reference).*

While in depth, multiparameter characterization and quantification of SGZ microglia would be interesting, we believe that it is outside the scope of this study. Due to overlapping somas and processes of microglia in this region, it is often difficult to resolve microglia in the SGZ as compared to the cortex where cell bodies are typically distant from one another. Our focus for this study remains the observation of transcriptomic differences between myeloid cells from hippocampi.

*4.- Supplementary Figure 2 would benefit greatly from a reorganization showing a single graph for each variable (read transcripts, unique genes, % of mito genes) showing side-by-side (with the same scale on the Y-axis) data (violin) for each library.*

We have re-scaled the violin plots so that plots have the same range for the Y-axis across runs.

*5.- The quality of the figures should be improved (e.g., homogenize the font; increase the font size (e.g., GO terms in the graphs in Fig3C, Fig4E; axis label on the bar in Fig.1C, Fig.1D, ); remove unreadable gene names in the volcano graph shown in Fig.3A,).*

We have homogenized font style to Arial and increased figure subpanel sizes to increase font size without crowding images. Additionally, as requested we have changed the labels to highlight genes with the highest statistically significant changes in expression.

*6.- Methods section. Single-cell RNA-seq Preprocessing and Alignment. Indicate that different versions of CellRanger were used for distinct replicates.*

I have clarified this by editing the text to “Preprocessing of the transcriptomic data was performed using CellRanger version 3.0.2 for the first sample and version 3.1.0 for the subsequent two samples using default parameters” in lines 189-191 of the manuscript.

*7.- Methods section. Microglial isolation. Please, indicate the route of administration of Tamoxifen (intraperitoneal, i.p.?).*

I have adjusted the manuscript to reflect this in line 120. “Seven-week old dual reporter mice were injected with Tamoxifen (100mg/kg) intraperitoneally once a day for four consecutive days.”

*8.- The manuscript needs a thorough revision as there are many errata (lack of spacing between words, duplication of "PMC" in many references in the reference section (e.g., PMCPMC4148642)). Error in the legend of Suppl. Fig.3: there is no panel D in the figure. Revise the integration of figures within the text as there are errors (e.g., Suppl. Fig. 2...).*

These errors have been corrected by removing the extra “PMC”. We have fixed the caption mistakenly labelled as Supplemental Figure 3D in the figure as it is a duplicate of Supplemental Figure 4D.

6. PLOS authors have the option to publish the peer review history of their article (what does this mean?). If published, this will include your full peer review and any attached files.

If you choose “no”, your identity will remain anonymous but your review may still be made public.

Do you want your identity to be public for this peer review? For information about this choice, including consent withdrawal, please see our Privacy Policy. No
Confidential to Editor

1. Do you have any potential or perceived competing interests that may influence your review? Please review our Competing Interests policy and declare any potential interests that you feel the Editor should be aware of when considering your review. If you have no competing interests, please write "I have no competing interests." I have no competing interests.
2. Did you receive any assistance in preparing this review (e.g. from a post-doc or graduate student)? If yes, please include their name below.
3. If accepted, do you think this submission should be highlighted on the PLOS ONE website? PLOS ONE does not evaluate manuscripts based on perceived significance or readership. We aim to provide tools for readers to filter and evaluate our publications. (optional) No
Do you want to get recognition for this review on Publons?
If you opt in, your Publons profile will automatically be updated to show a verified record of this review in full compliance with the journal’s review policy. If you don’t have a Publons profile, you will be prompted to create a free account.

Yes

1.	Parkhurst CN, Gan WB. Microglia dynamics and function in the CNS. Curr Opin Neurobiol. 2010;20(5):595-600. Epub 2010/08/14. doi: 10.1016/j.conb.2010.07.002. PubMed PMID: 20705452; PubMed Central PMCID: PMCPMC3708473.

2.	Lawson LJ, Perry VH, Dri P, Gordon S. Heterogeneity in the distribution and morphology of microglia in the normal adult mouse brain. Neuroscience. 1990;39(1):151-70. Epub 1990/01/01. doi: 10.1016/0306-4522(90)90229-w. PubMed PMID: 2089275.

3.	Elmore MR, Najafi AR, Koike MA, Dagher NN, Spangenberg EE, Rice RA, et al. Colony-stimulating factor 1 receptor signaling is necessary for microglia viability, unmasking a microglia progenitor cell in the adult brain. Neuron. 2014;82(2):380-97. Epub 2014/04/20. doi: 10.1016/j.neuron.2014.02.040. PubMed PMID: 24742461; PubMed Central PMCID: PMCPMC4161285.
