# Supplementary material for: Distinct microglial transcriptomic signatures within the hippocampus
Source: PLoS One. 2024 Jan 5;19(1):e0296280. doi: 10.1371/journal.pone.0296280 (PMC10775894; doi:10.1371/journal.pone.0296280)
Supplement: Attachment — Submitted filename: Response to reviewers_August_sk.docx [file pone.0296280.s012.docx]

PONE-D-23-16545

Unique Microglial Transcriptomic Signature within the Hippocampal Neurogenic Niche

PLOS ONE

Reviewers' comments:

Reviewer's Responses to Questions

**Comments to the Author**

1. Is the manuscript technically sound, and do the data support the conclusions?

The manuscript must describe a technically sound piece of scientific research with data that supports the conclusions. Experiments must have been conducted rigorously, with appropriate controls, replication, and sample sizes. The conclusions must be drawn appropriately based on the data presented.

Reviewer #1: Partly

Reviewer #2: Partly

2. Has the statistical analysis been performed appropriately and rigorously?

Reviewer #1: I Don't Know

Reviewer #2: Yes

3. Have the authors made all data underlying the findings in their manuscript fully available?

The [PLOS Data policy](http://www.plosone.org/static/policies.action#sharing) requires authors to make all data underlying the findings described in their manuscript fully available without restriction, with rare exception (please refer to the Data Availability Statement in the manuscript PDF file). The data should be provided as part of the manuscript or its supporting information, or deposited to a public repository. For example, in addition to summary statistics, the data points behind means, medians and variance measures should be available. If there are restrictions on publicly sharing data—e.g. participant privacy or use of data from a third party—those must be specified.

Reviewer #1: Yes

Reviewer #2: Yes

4. Is the manuscript presented in an intelligible fashion and written in standard English?

PLOS ONE does not copyedit accepted manuscripts, so the language in submitted articles must be clear, correct, and unambiguous. Any typographical or grammatical errors should be corrected at revision, so please note any specific errors here.

Reviewer #1: Yes

Reviewer #2: Yes

5. Review Comments to the Author

Please use the space provided to explain your answers to the questions above. You may also include additional comments for the author, including concerns about dual publication, research ethics, or publication ethics. (Please upload your review as an attachment if it exceeds 20,000 characters)

*Reviewer #1: Chintamen et al., explore the diversity of the myeloid cells in the hippocampus, which are known to be extremely heterogenous both in morphology and gene expression profile. They enrich for myeloid cells utilizing a tdTomato construct, applying single-cell RNA sequencing on 12 mouse hippocampi, and revealing a rare microglia subpopulation with high Cd68 expression and, possibly, specific Cd63 expression (cluster 8). Indirect evidence of immunohistochemistry of cluster 8 markers, suggests this subpopulation is localized in dentate gyrus subgranular zone. Cluster markers are a mix of mostly DAM genes and some genes suggesting neurogenesis and synaptic pruning. The last figure aims to prove that the morphology of the SGZ microglia is distinct from homeostatic microglia and that cluster 8 is transcriptionally similar to DAMs. This study reports a highly relevant findings for any scientist working on the hippocampus and raises an extremely interesting point of SGZ specialized microglia. The efforts that are made to connect a transcriptional single-cell cluster to a biological function in the SGZ is appreciated. However, especially for figure 3 and 5, which are conceptionally the most interesting, I have major concerns to address:

Figure 5A-C: The argument made in figure 5A is that microglia morphology in the dentate gyrus are different from microglia in the cortex. This is quite unsurprising and does not support the following claim, which contains a comparison between hippocampal microglia:
‘These results are in accordance with transcriptome analyses that suggest microglia display an alternative phenotype in the neurogenic niche.*

We thank the reviewer for the feedback. While the majority of our manuscript focuses on differences between transcriptionally defined clusters within the hippocampus, our intention with Figure 5 is to highlight the differences between SGZ microglia and well characterized microglia. As cortical microglia have been most characterized, we sought to specifically compare the traits of our cluster 8 microglia with these more well characterized microglia. The genesis for this study was from the observation that SGZ microglia which are located in a clear spatially confined niche have very different properties from cells traditionally thought to be homeostatic microglia. The fact that they have in a resting state properties and marker gene expression similar to those found in disease is important to point out because this reflects regional heterogeneity, which is not well understood in the adult brain. For this reason, we took a molecular approach to characterizing the cells within the hippocampus. Since cortical microglia are highly represented within whole brain homogenates and have been examined by others such as Hammond et. al. 2019, Li et. al. 2019, we excluded them from the RNA seq experiments for this study.

*In short, the claim is about differences within the hippocampus, the plots are comparing cortex and dentate gyrus. Figure 5 is also not convincingly proving a phagocytic function of SGZ microglia. In my opinion, Fig 5A-C have no relevance regarding the message about hippocampal diversity in myeloid cells and would be more suitable to place in a supplement.
It would be highly interesting and valuable to instead analyze morphology in figure 2 images (tdTomato) for the different hippocampal zones (SGZ, GL, GM). Direct evidence for altered morphology in the SGZ compared to the GL and GM would be a convincing argument that supports the claim.

Figure 5E: The results from integrated keren-shaul and hippocampal microglia are a valuable addition to this paper adding a relevant perspective on what a “DAM” signature actually represents. However, I was struggling to understand the integrated UMAP presented in figure 5E and relate it back to figure 1D. A plot is missing that shows the distribution of cluster 8 microglia (potential SGZ microglia) in the integrated data. The only message figure 5E is currently conveying, is that the abundance of cluster 7, 9 and 10 is altered between the two datasets. There are no abundance differences visible in cluster 5.

In short, the claim in line 468: “found that microglia exclusively from cluster 8 with DAM” is not supported.
Cluster 5 seems to be the DAM phenotype, based on the marker expression in figure 5F, however, it is not shown how integrated cluster 5 (DAM) is linked to original cluster 8 (hippocampal SGZ microglia). My suggestion is that the authors could show the UMAP with all integrated data but color the cells as non-cluster 8 (grey) and cluster 8 (red) to indicate the hippocampal SGZ microglia. It is essential to visualize the abundance and distribution of the cluster 8 “SGZ” microglia in the integrated data.*

We appreciate these insights and agree that it is important to visualize the abundance and distribution of these microglia. We have added description in the text to better explain the motivation for the morphological characterizations. Qualitatively, one can observe consistent and prominent differences in the SGZ. The presence of a subset of cells that appeared to have an alternative morphology compared to cortical microglia is what motivated us to directly compare results to a study that specifically examine a distinct phenotypic state. Since cortical microglia are in high abundance and likely dominate the results for characteristic traits in homeostatic microglia, we chose to compare SGZ microglia with cortical microglia for morphological characterization. In order to clarify the rationale for these comparisons to cortical microglia, we have included additional text in lines 576-581 (see below).

“As the cortex is the largest region of the murine brain, microglia from this region represent the highest number of any one region. While the distribution of microglia in the resting cortex is characterized as tiled with microglial branches from adjacent cells maintaining distinct, non-overlapping territories, some regions of the brain do not have this patterning, particularly in regions with high neuronal densities [1, 2]”

We have also now provided a correlation matrix in supp fig 8 to show that the cluster 5 in the integrated dataset corresponds to cluster 8 from figure 1D. The UMAP in figure 5E is a representation of the integrated dataset split by Hippocampal microglia and Keren-Shaul data to show the DAM signature via the cluster 5. Additionally, we have now included the plot with cells from cluster 8 highlighted which the reviewer has asked for in Figure 5D.

*Figure 3: The volcano plot in figure 3A clearly illustrates the point that cluster 8 is expressing DAM genes. The top GO terms should be microglia-related, such as innate immune response , antigen presentation, and cytokine production. The fact that only a selection of GO terms related to neurons is shown, while omitting the top GO terms that are most significant, is misleading.
I would strongly suggest to explain when and why a selection of GO terms is displayed. It could be improved by showing both the most significant GO term enrichment and the ‘cherry-picked’ GO terms to make it clear to the reader. However, the finding does raise an interesting scientific point.. which genes are part of GO term “neurogenesis”, “neuron development”, “synapse organization” ( I did not find this in S Table 2??) and are these genes specific to neurons? Where are they in the volcano plot? Is it possible these genes are not expressed by microglia at all; this would suggest that microglia phagocytose apoptotic and immature neurons in the SGZ and that neuron-derived RNA is detected in your microglia transcriptome. Reports using RiboTag suggest this could be an explanation (PMID: 29777220) and could be used to extend the argument made in line 417-422. Some neuronal genes could be added to figure 3C.
Optional, out of interest, neuronal RNA in the differential gene list could be further investigated using a simple webtool* [*https://www.syngoportal.org/*](https://www.syngoportal.org/) *.*

We appreciate this feedback. To ensure we add more clarity for readers, we have explicitly stated that we have highlighted processes relating to neurogenesis and immune responses in Figure 3B (lines 495-501) and Figure 5D (570-574), respectively. In addition, we also explicitly point readers to the full list of GO terms which is included as supplemental table 2, where they can also refer to the information for specific terms such as “neurogenesis” Neurogenesis (GO:0022008), “neuron development” – (GO:0048666), “synapse organization” (GO:0050808), “axon development” (GO:0061564), “generation of neurons” (GO:0048699), “neuron differentiation” (GO:0030182), “neuron projection development” (GO:0031175), and etc.

We also thank the reviewer for pointing us to the “syngo portal” as a useful resource. We tried to use this tool on our most differentially expressed genes. However, these were not in the database as the reviewer pointed out this database is for neuronal gene enrichment. The top differentially expressed genes are exclusively expressed in microglia. Their annotation as genes enriched in the context of neurogenesis likely is due to the upregulation of these genes during processes associated with embryonic and early postnatal development of the nervous system.

*Figure 1C: Please do NOT show fluoresence intensity, as it is very hard to compare the different antibody results. Please DO show the percentage of cells that is positive for Tmem119 (and other markers) and tdTomato in comparison to a control. How many cells are triple positive for Tmem119+CD45+CD11b+ and how many cells are quadruple positive Tmem119+CD45+CD11b+tdTomato+?
Negative controls using unstained cells, single staining controls and fluorescent minus one (FMO) controls are not shown. These are important for setting parameters of true positive and negative cell populations. Otherwise, if lacking, please show ungated populations that serve as “negative” cells (e.g. non-microglia cells should be negative for CD45).*

Thank you for these suggestions. As suggested, we now represent the information in supplemental figure S1B-S1C regarding the overlap between TdTomato cells and the cell surface markers (Tmem119, Cd11b/Cd45). We have also now included data from unstained samples, single stained samples, and samples with isotype controls. These include percentages of cells with dual expression or triple expression after gating on fluorescence levels.

Due to limitations of how many channels we had available, we were not able to stain for Tmem119 and Cd11b in the same flow samples. However, the flow samples originated from the same homogenates so percentages of cells expressing these respective marker genes can be directly compared.

Minor comments:
*Line 60-61 versus line 71-76: please be consistent on terms “microglia”, “myeloid lineage cells” and “immune cells”. Could the authors make an effort to distinguish microglia, CNS-associated macrophages, and infiltrating immune cells in the introduction? For example, in line 71 it appears to me microglia are grouped under immune cells whereas in line 60 microglia are specifically not immune cells.*

We recognize that without an explanation of these terms it is difficult to understand the distinction between these cell populations. Thus, we have changed the text in line 60 and added the text listed below (lines 66-75) to describe that macrophages come from microglia in the introduction.

“Under resting conditions, the immune compartment of the central nervous system (CNS) is comprised of myeloid lineage cells, microglia and other macrophages which contain distinct transcriptomic and phenotypic properties [11, 12]. The latter of the two are typically found in the meninges, perivascular regions, and choroid plexus [13]. Collectively, these non-microglial macrophages are termed CNS-associated macrophages (CAMs) or Border Associated Macrophages (BAMs) [11, 12]. However, as microglia are the primary macrophage in the brain parenchyma and found to be actively interacting with neurons, they are more widely studied and characterized compared to macrophages in non-parenchymal tissue, particularly in the context of neurodevelopmental processes.”

*Line 226: methods: Why was Harmony used for the data integration with the Keren-Shaul dataset, whereas previously (line 189) Seurat’s IntegrateData with CCA was used to integrate samples and correct technical effects? What is the rationale for this different methodology? Harmony strongly alters the gene expression information and clustering results might be even more overlapping if the CCA approach is used for the Keren-Shaul study.*

The reason for choosing Harmony to integrate our dataset with Keren-Shaul’s dataset is the ability of Harmony to correct for multiple experimental/technical factors. Harmony projects cells into a shared embedding in which cells group by their cell types rather than dataset-specific conditions and it corrects for multiple experimental factors which was the case for our integration step since both datasets have different technical factors and tissue sources. Harmony (https://www.ncbi.nlm.nih.gov/pmc/articles/PMC6884693/) specifically mentions that it does not alter the expression values of individual genes to account for dataset-specific differences.

*Line 339: Clusters 4,5,6, 7 and 11 failed to show enrichment of at least ten nuclear genes. This raises the concern this single-cell dataset is a bit overclustered. However, I understand it cannot be changed at this point in the project. Cluster 4 expresses mitochondrial-derived markers, suggesting this is a stressed, less viable cluster of cells that should be removed. Cluster 5 and 6 are appropriately addressed in the text. In my opinion cluster 7 and 11 could be merged to one cluster. This is sometimes done in single-cell analysis to revert the overclustering (It can be done by simply giving them the same label in cluster annotation using Seurat::RenameIdents()).*

We thank the reviewer for these suggestions. All cells that are present in the initial clustering (Fig 1D) have passed the QC filters and therefore these cells express an acceptable number of mitochondrial genes. We intentionally chose to retain a higher number of clusters so as not to obscure or confound any cell states or subpopulations that may be identified in future studies. However, we recognize that these populations may also potentially represent cells that are transitioning towards states where their quality may be potentially compromised. Hence, we focused our downstream analyses in Figures 3 and 5 on cells derived from cluster 8 versus clusters from homeostatic clusters (now labeled 12 and 13). We also appreciate the reviewer’s feedback and suggestions on how to merge clusters. Upon receiving this feedback, we agree that clusters 7 and 11 should be merged. We have thus updated all main text figures and supplemental figures to reflect these changes. Note, we have also changed the supplemental tables including the differential gene expression analysis between clusters to reflect this.

*Line 408: incorrect reference, should reference supplemental figure 7*

We had rearranged supplemental figures 6 and 7 in response to previous reviewers’ feedback on the order of these figures. However, the supplementary figures may not have been updated upon our resubmission. We thank the reviewer for their vigilance in noting this discrepancy.

*Supplemental figure 4: the heatmap is very blurred.*

We apologize for the lack of resolution in this figure. We have reattached and updated version incorporating changes in clustering (see above for merging of clusters 7 and 11).

*Figure 1A: this is outside the scope of the current study, but relevant for follow-up work using the same reporter. Did you check tdTomato signal in mice without a TAM injection? Unfortunately, Rosa26-floxP-tdTomato constructs can be leaky due to the short stop codon, please refer to PMID: 32125704 “STOP floxing around: specificity and leakiness of inducible Cre/loxP systems” to verify the validity of your strain. For the main message and findings of this paper I do not believe it matters.*

We checked tdTomato expression with Tamoxifen injection compared to vehicle injections. We noted tdTomato expression in some cells. These appeared to all have microglial morphology, thus leading us to believe there is some low-level leaky tdTomato expression.

*Please reconsider the manuscript title “Unique Microglial Transcriptomic Signature within the Hippocampal Neurogenic Niche”, since the single-cell data does not prove the microglia cluster 8 is unique to the SGZ nor is there sufficient evidence from immunohistochemistry to localize it to the neurogenic niche. A more suitable alternative could be “Distinct microglial transcriptomic signatures within the hippocampus”*

We appreciate the reviewer’s views on how best to capture the results of our manuscript. We have changed the title to reflect a more accurate and specific summary of our finding and have changed the title of our manuscript to the reviewer’s suggested title.

*Reviewer #2: Chintamen et al., used single-cell RNAseq to explore the transcriptional profiles of myeloid lineage cells isolated from the hippocampus. The authors noted microglial heterogeneity and highlighted a unique population (cluster 8) of microglia that demonstrate higher level of phagocytosis-related genes. Based on histological analysis of selected markers, the authors have attributed this population of microglia to a neurogenic niche in the subgranular zone of hippocampus dentate gyrus.*

Major concern
*1. While the authors have cited other single-cell RNAseq studies on microglia, they have not rigorously compared their results to these studies. I appreciate that they have compared it with Keren-Shaul et al. (2017). However, from what I can gather from their differential gene expression tables, their cluster 8 is also very similar to an early postnatal microglia population described by Hammond et al. 2019 (cluster 3).*

We thank the reviewer for the keen observation that our cluster 8 bears resemblance to cluster 3 in Hammond et. al. We created a Venn diagram to illustrate the overlap of the genes enriched between cluster 8 in our manuscript with the early postnatal microglia. This can now be found as Supplemental Figure S8B. We investigated some specific genes that are present in both lists and highlighted their presence by our cluster 5 from in the integrated dataset which we have included in the manuscript as Supplemental Figure S8D.

Additionally, we have also included a Venn diagram (Supplemental Figure S8C) showing overlap between enriched genes in our Cluster 8 and the genes upregulated in cluster 3 from the Hammond et. al. dataset and incorporated language to describe similarities and differences between these enriched genes in these datasets.

*2. Similarly, in line 374 and 375, the authors mentioned that a ribosomal enriched cluster that has been reported but not characterized, but the observation has not only been reported by multiple studies, it has been reviewed (*[*https://www.ncbi.nlm.nih.gov/pmc/articles/PMC8225243/*](https://www.ncbi.nlm.nih.gov/pmc/articles/PMC8225243/)*). These are just some of the existing literature that I was able to identify with relatively few searches. Along with the lack of mentions of bulk-RNAseq work that highlighted microglial heterogeneity across the brain, my main concern is whether other information and links to existing work were also missed and that the authors have overstated their findings due to insufficient comparison to existing work.*

We thank the reviewer for pointing out the literature that has emerged for ribosomal gene enriched clusters which were not published when we first conducted these experiments. We have included the reference to the review mentioned as well as a couple of other papers and included the description bellow in lines 468-475 of the main text.

“Cluster 10 exhibits increased expression of genes associated with encoding ribosomal protein subunits (genes with *Rps* and *Rpl* as their prefix) and also contains the highest *ApoE* expression. This group of cells undergoing high metabolic activity has been identified by other groups and may reflect cells with more metabolic needs which are typically enriched with immune reactivity [3-5].”

We have referenced Kreisel et al 2019 which was a bulk RNAseq data set comparing regional differences between microglia from the dentate gyrus (DG) vs CA1. In addition, we have also alluded to the work by Artegiani et al, Hammond et al, and Li. et al which do use single cell rnaseq data but do not enrich for microglia (in the case of Artegiani et al) or do not enrich for microglia in specific regions. We have also referenced Ayata et al, which looks at regional heterogeneity in their main text with an emphasis on cerebellar microglia.

*3. If the authors want to keep clusters with upregulation on mitochondrial genes and not regress out cell cycle genes, they should really include more quality control figures (i.e. plots showing nCount_RNA, nFeature_RNA, % ribosomal RNA, % mitochondrial RNA, cell cycle scoring (you can get that from using CellCycleScoring in Seurat). It is difficult to review and assess their quality control.*

nCount_RNA, nFeature_RNA, and % mitochondrial RNA are presented as “Transcript reads”, “unique genes”, “%mito genes” respectively. In addition, as per the reviewer’s suggestion we have added % ribo genes as a violin plot along the previously described QC plots in Supplemental Figure S2A. We have also added a UMAP plot showing the cell cycle classification of each of the cells in Supplemental Figure S2B. We noted that this analysis has been developed with human genes in mind. We converted the list of human genes used in the “Cell Cycle Scoring vignette” (<https://satijalab.org/seurat/articles/cell_cycle_vignette.html>) and converted these genes into their orthologous murine equivalent. For this, we used BiomaRt as suggested on some seurat forums (https://github.com/satijalab/seurat/issues/2493). We do find that not every gene has an ortholog in the mouse genomic reference which may potentially skew the scoring results.

*4. While I do not believe Cluster 8 is doublet and the authors have included a figure showing the lack of clusters demonstrating doublets in their response to other reviewers, I have two issues with the manuscript in its current form. 1) Even if there isn’t a significant pattern in doublets, the figure shown to the reviewer should be included in the supplementary. It should also be demonstrated that the removal of doublets identified by Doubletfinder does not significantly improve the cluster separation. 2) Doubletfinder works by averaging the transcriptional profiles of randomly selected cell pairs. If there were to be doublet in this dataset, part of the doublet pair could be neuronal. However, as the cells were FACs sorted, the majority of the neuronal cells would not be present in the dataset, therefore Doubletfinder would not be able to generate a good representation of doublet transcriptional profiles. In order to more accurately remove doublets in this dataset, you would need integrate a single cell RNAseq dataset with non-FACS sorted, then carry out doublet detection, remove all doublet and then keep the singlet cells from this study. I suspect doublets might be the source of the small fraction of expression of other cell type markers indicated in line 309-311. While I don’t expect this level of doublet removal, I do not think the response to Reviewer 2’s comment 4 was sufficient and at least 1) should be incorporated in the manuscript.*

Thank you for your acknowledgement regarding cluster 8. To answer the point raised here, we investigated the doublets in our data by using DoubletFinder (2.0.3). A total of 1108 cells (~6% of total cells) were predicted to be doublets based on DoubletFinder. We highlighted these cells on a UMAP and it seems like the chances of clusters to be rearranged/improved is highly unlikely even if we remove these cells given these cells are not confined to any specific cluster as well as are low in number. We have incorporated this figure as Supplemental Figure S2C in the manuscript as suggested and included an explanation in lines 374-385 of the manuscript .

*5. It would be useful to see Allen Brain atlas showing the Cluster 8 upregulated gene expression in other brain regions to see if they are specific to the hippocampus region.*

We have added images from the Allen Brain atlas showing coronal sections of these genes.

*6. The rational of selecting 20 PCs is unclear – is a Jackson straw plot or elbow plot made to determine this number?*

We determined this based on an elbow plot which we have now included as a supplementary figure S3C and referenced in lines 239-240.


Minor concerns
*1. It is not clear to me what clusters Figure 5E are referring to and how they relate to the main clustering in this study. The comparison to Keren-Shaul et al’s work is generally not well described and readers cannot easily follow the rationale.*

Given the differences in transcriptomic signature between cluster 8 and homeostatic microglia characterized by several groups, we sought to understand the functional role these cells may potentially have. When examining the list of genes found to be enriched in our cluster, we noted that many of them were also similar to that published by Keren-Shaul et. al. We found this observation interesting given that these are cells in a healthy brain. In order to classify whether these cells are actually DAM-like beyond some genes, we performed integration analysis which showed us that these cells are more similar to DAM than they are to homeostatic microglia. We have expanded our explanation and rationale of these analyses and data in lines 594-694.

*2. Line 530 – citation style change*

Thank you for pointing out this discrepancy, we have fixed the citation notation.

*3. Line 441 – did you mean Supp Figure 7?*

The ordering of the supplemental text was rearranged after the first round of revision. We had swapped the confocal images and images from the Allen brain atlas and have kept the reference to Supplemental Figure 6. However, a previous version of our supplemental file was inadvertently attached to our manuscript submission. Thank you for your attention to this.

*4. Figure 1E and 5F – the macrophage clusters should be removed from these figures (and placed in supplementary) to allow the colour scale to reflect a wider range of gene expression.*

For Figure 1E we have intentionally kept all clusters in the figure in order to identify cell identity across various clusters. Further analyses in this study focus on expression levels between microglial subpopulations cluster 8 and homeostatic clusters 12 and 13 with cell expression displayed as violin plots which do capture the full range of expression of homeostatic and upregulated genes (see figure 3C).

Figure 5F on the other hand does not consist of macrophages. The cells we included for this integration analysis were clusters 12 and 13 as homeostatic microglial cells along with cells from cluster 8. Cells that were integrated from the Keren-Shaul dataset were specifically cells expressing microglial marker genes only and hence excluded any non-microglial macrophages. In order to highlight the scale for DAM genes within the non-integrated cells in homeostatic microglial clusters 12 and 13 alongside cluster8, we have selectively plotted gene expression of these genes in Supplemental Figure 8A.

1.	Parkhurst CN, Gan WB. Microglia dynamics and function in the CNS. Curr Opin Neurobiol. 2010;20(5):595-600. Epub 2010/08/14. doi: 10.1016/j.conb.2010.07.002. PubMed PMID: 20705452; PubMed Central PMCID: PMCPMC3708473.

2.	Lawson LJ, Perry VH, Dri P, Gordon S. Heterogeneity in the distribution and morphology of microglia in the normal adult mouse brain. Neuroscience. 1990;39(1):151-70. Epub 1990/01/01. doi: 10.1016/0306-4522(90)90229-w. PubMed PMID: 2089275.

3.	Zhan L, Fan L, Kodama L, Sohn PD, Wong MY, Mousa GA, et al. A MAC2-positive progenitor-like microglial population is resistant to CSF1R inhibition in adult mouse brain. Elife. 2020;9. Epub 2020/10/16. doi: 10.7554/eLife.51796. PubMed PMID: 33054973; PubMed Central PMCID: PMCPMC7591254.

4.	Tansley S, Uttam S, Urena Guzman A, Yaqubi M, Pacis A, Parisien M, et al. Single-cell RNA sequencing reveals time- and sex-specific responses of mouse spinal cord microglia to peripheral nerve injury and links ApoE to chronic pain. Nat Commun. 2022;13(1):843. Epub 2022/02/13. doi: 10.1038/s41467-022-28473-8. PubMed PMID: 35149686; PubMed Central PMCID: PMCPMC8837774.

5.	Mendes MS, Majewska AK. An overview of microglia ontogeny and maturation in the homeostatic and pathological brain. Eur J Neurosci. 2021;53(11):3525-47. Epub 2021/04/10. doi: 10.1111/ejn.15225. PubMed PMID: 33835613; PubMed Central PMCID: PMCPMC8225243.
