# Supplementary material for: Distinct microglial transcriptomic signatures within the hippocampus
Source: PLoS One. 2024 Jan 5;19(1):e0296280. doi: 10.1371/journal.pone.0296280 (PMC10775894; doi:10.1371/journal.pone.0296280)
Supplement: Attachment — Submitted filename: Thank you for submitting your revised manuscript.docx [file pone.0296280.s013.docx]

Thank you for submitting your revised manuscript. I have reviewed it with great interest and observed several improvements. I am happy to accept this paper. Before finalizing the article, I would like to propose three minor modifications to the authors.

# Minor details:

Line: 499 your heading of the paragraph is missing a word in track changes: "SGZ microglia display morphology and gene expression profiles **that** deviate from a more homeostatic phenotype."

Supplemental figure 4: the legend can be adjusted to “scaled expression” (expression of -2 is not possible so I assume this is a z-score , scale = TRUE setting somewhere).

The phrase “resting brain” and “resting cortex” is used in multiple instances in the manuscript. It sounds a bit awkward, you might want to consider “healthy brain” “under control conditions” or otherwise.
